# Incivility experiences of racially minoritised hospital staff, consequences for them and implications for patient care: An international scoping review

**DOI:** 10.1111/1467-9566.13760

**Published:** 2024-03-20

**Authors:** Olivia Joseph, Ghazala Mir, Beth Fylan, Pam Essler, Rebecca Lawton

**Affiliations:** ^1^ Faculty of Medicine and Health School of Psychology University of Leeds Leeds UK; ^2^ National Institute for Health Research (NIHR) Yorkshire and Humber Patient Safety Research Collaboration Bradford UK; ^3^ Faculty of Medicine and Health Leeds Institute of Health Sciences University of Leeds Leeds UK; ^4^ Faculty of Life Sciences School of Pharmacy and Medical Science University of Bradford Bradford UK

**Keywords:** healthcare workers, quality of care, scoping review, workforce equity, workplace incivility

## Abstract

Workplace incivility is a pervasive complex problem within health care. Incivility manifests as subtle disrespectful behaviours, which seem inconsequential. However, evidence demonstrates that incivility can be harmful to targets and witnesses through negative emotions, poorer mental health, reduced job satisfaction, diminished performance and compromised patient care. It is unclear to what extent existing research critically explores how ethnicity, culture and racism influence how hospital staff experience incivility. This global scoping review systematically analysed existing research exploring the specific ways incivility manifests and impacts racially minoritised hospital workers. Of 2636 academic and 101 grey literature articles, 32 were included. Incivility experiences were categorised into four themes: (1) Cultural control, (2) Rejection of work contributions, (3) Disempowerment at work and (4) Managerial indifference. The included articles highlighted detrimental consequences, such as negative emotions, silencing, withdrawal and reduced support‐seeking behaviours. Few studies presented evidence regarding the negative impacts of incivility on patient care. Racialisation and racial dynamics are a significant factor for hospital‐based incivility. Currently we do not know the extent to which racialised incivility is associated directly or, perhaps either via burnout or disengagement, indirectly with poorer care. This knowledge can inform the creation of comprehensive, evidence‐based interventions to address this important issue.

## INTRODUCTION

Workplace incivility is a significant, persistent and increasing concern within public institutions, and healthcare is no exception (Keller et al., 2020; O’Daniel & Rosenstein, 2008; Westbrook et al., 2020; The Joint Commission, 2021). Within acute, high‐risk interdisciplinary hospital teams, teamwork and effective communication is essential to ensure care is safe. Despite this, up to 90% of health‐care workers report frequent exposure to uncivil behaviours (Credland & Whitfield, 2021). Workplace incivility refers to subtle covert behaviours within the workplace that violate established norms for mutual respect with unclear intentionality, which commonly manifests through rude, disrespectful and discourteous behaviours (Andersson & Pearson, 1999). Experiencing or witnessing uncivil behaviours, such as gossiping, eye rolling and being ignored have been shown to negatively impact the mental health, wellbeing (Credland & Whitfield, 2021) and clinical decision‐making of hospital workers (Bradley et al., 2015). Research shows that incivility poses a serious risk to communication, information seeking and sharing between colleagues, damaging collegial relationships and ultimately eroding the quality and safety of care provision for patients (Katz et al., 2019; Riskin et al., 2015). Organisational consequences occurred through lower job satisfaction, reduced organisational commitment and higher turnover intentions (Han et al., 2021; Martin & Zadinsky, 2022). These findings suggest that hospital workers who experience uncivil behaviours can affect information flow through harmful effects to the individual, workgroup, work related outcomes and patients via care processes.

The theory of workplace incivility is characterised by four key features: (1) involving two or more social actors, (2) low‐intensity subtle behaviours, (3) ambiguous intent and (4) the social context (Andersson & Pearson, 1999). Incivility is a subjective assessment; therefore, an individual perceives that mutual respect has been violated through the subtle actions or inaction of another person. Individuals perceive behaviours in a variety of ways and this can be influenced by several factors, including the status, position, experience or cultural background of the individual, as well as the social context (Cortina et al., 2017; Schilpzand et al., 2014). Certain demographic characteristics have been shown to increase the likelihood of exposure to incivility in health care, such as gender, age, tenure, rank and ‘race’, albeit the evidence is conflicting (Cortina et al., 2017; Schilpzand et al., 2014). Two recent studies conducted a meta‐analysis of over 20 years of incivility research across several industries (including health care), both finding that ‘race’ and ethnicity are antecedents of incivility experiences (Han et al., 2021; Yao et al., 2022). This is interesting because an important critique of the generalised incivility literature highlights the missed chance to account for the influence of social systems of oppression, such as racism and sexism, which influence drivers and perceptions of incivility. With the increasing legislation and societal intolerance to overt explicit prejudicial behaviour, Cortina (2008) suggests that incivility may be a route for pervasive forms of discrimination to persist, as they do not attract the same penalties or repercussions as overt racial discrimination. In summary, whilst there is evidence, albeit conflicting, that people from a minoritised ‘race’, ethnicity or language are more likely to be targets of incivility, the evidence is weak and limited.

Racism is defined as ‘a system of structuring opportunity and assigning value based on the social interpretation of how one looks (so called “race”), that unfairly disadvantages some individuals and communities, unfairly advantages other individuals and communities and saps the strength of the whole society through the waste of human resources’ (Jones, 2018, p. 231). This social power system is historically influenced and embedded in human behaviours, including racial incivility, providing those racialised as White Europeans with advantages and privileges, which are ideologically and materially reinforced through disproportionate allocation and distribution of resources (Jones, 2018, Ray, 2019). ‘Race’ is often perceived as an inherent biological trait; however, it is more accurately a socially and culturally constructed personal characteristic, laden with meaning (Milner & Jumbe, 2020). The cultural meanings include associated stereotypes, attitudes and prejudices related (but not limited to) value, ability and intellect, imposed through processes of racialisation.

Within organisations, Ray (2019) theorises that racial dynamics are interplayed across micro, meso and macro levels extending beyond ‘race’ as an identity or individual behaviours of ‘bad actors’ to racialised organisations which are ordered through hierarchies, logics and processes and wider societal institutions (e.g. law, media and politics). These racialised practices become normative, hidden and invisible enablers of mechanisms that uphold racial inequity, suggesting that the very structure and functioning of an organisation can be shaped by racialised dynamics that influence behaviours and interactions (Ray, 2019). As such, we need to better understand how incivility affects racially minoritised^1^ staff through racialised dynamics that are embedded in how an organisation operates, makes decisions, defines values, activities and workflows. These dynamics reveal the complexities of how the physical, psychological and emotional work environments are shaped by and influence uncivil interpersonal interactions that are underpinned by mechanisms of racialisation.

Drawing from organisational, social psychology and discrimination literature, and shaped by theories, such as aversive racism (Dovidio & Gaertner, 2000), cultural racism (Appiah, 1992) and microaggressions (Sue & Constantine, 2007), Cortina et al. (2011) empirically tested the theory of ‘selective incivility’ across public sector organisations, including the city government, law enforcement and the United States (US) military. This theory proposes that individuals hold negative racial biases which manifest through prejudice (an underlying subconscious mood or emotion termed affect), stereotypes (a fixed generalised judgement or belief that imposes an oversimplified characteristic to a group of people) and subtle behaviours (covert discriminatory acts) towards marginalised groups. They suggested that in certain circumstances, generalised incivilities are driven by conscious or unconscious biases that selectively target racially minoritised individuals. In addition to racialised dynamics, gender and class are foundational in organisational theory, yet are often overlooked in terms of the multiple effects of co‐occurring structural inequities. Scholarly attention to ‘intersectionality’ acknowledges that racism, sexism and classism can have multiplicative effects, shaping specific patterns of disadvantage (Crenshaw, 1989; Ray, 2019; Smith et al., 2020), which can influence complex incivility experiences. Cortina et al. (2011) analysed selective incivility considering both gender and ‘race’, and found that women of colour were more likely to experience incivility compared to other groups. However, the interconnection between ‘race’ and gender remains inconsistent across studies (Ruvalcaba et al., 2018; Smith et al., 2020), indicating a need for further research into contextual factors, occupational roles and work activities influencing incivility interactions.

In socially hierarchal health‐care organisations, there are uneven distributions of power, social status and opportunities. Cortina et al. (2011) proposes that within subtle uncivil social processes, instigators can utilise their social status to bury or conceal the intention in an attempt to maintain an egalitarian image within the workplace. These findings are particularly important within the hospital context, due to the global rise in racially and ethnically diverse workforces and the persistent inequities that these workers face (Abubakar et al., 2022). For example, in the UK context, 24% of the NHS workforce self‐identify as Black, Asian and Minority ethnic. Despite this, they are under‐represented in senior positions and year on year they report experiencing significantly higher levels of bullying, harassment, abuse and discrimination (WRES NHS England, 2023). Given the empirical investigation of ‘selective incivility’ and its implications, these findings highlight the need for proactive efforts to handle covert discriminatory behaviours and promote genuine inclusivity, particularly in health‐care settings marked by increasing diversity and persistent disparities in staff and patient experience.

Literature examining racially minoritised experiences of incivility is largely found in other industries outside of health care, such as hospitality, retail service and governmental agencies (Cortina, 2008; Cortina et al., 2011; Kern & Grandey, 2009) or specifically focuses on or includes negative behaviours at the higher intensity end of the spectrum, such as bullying (Keller et al., 2020) and racism (Hamed et al., 2022). To our knowledge, no previous scoping review has focused on exploring racially minoritised workers incivility experiences and subsequent consequences in the hospital setting. Thus, this review maps current knowledge and gaps in evidence, which can support intervention development and the creation of holistic interventions to address incivility within the workplace.

## METHODS

### Aims

This scoping review aimed to describe the extent and breadth of current evidence concerning how racially minoritised hospital workers experience incivility and its associated consequences.

### Design

This study was conducted using the Joanna Briggs Institute six stage approach (Peters et al., 2020), which has been adapted from the original five stage version outlined by Arksey and O’Malley (2005). The six stages include (1) identifying the research question, (2) iterative development of the search strategy for identification of relevant studies, (3) selecting relevant studies, (4) extracting the data, (5) collating, summarising and reporting the results and (6) consultation with relevant stakeholders. The full protocol is registered with the Open Science Framework (osf.io/6t5c4). The reporting of this scoping review follows the PRISMA guidelines (Tricco et al., 2018), in addition to guidance with a particular focus on health equity (Welch et al., 2012).

### Stage 1: Identifying the research question

The first stage involves the development of appropriate, broad research questions to guide the identification and selection of appropriate academic and grey literature sources (Arksey & O’Malley, 2005). Three specific research questions were identified in collaboration with the review team and relevant stakeholders (including racially minoritised hospital staff and patients), these included: (1) How do racially minoritised hospital workers experience incivility and what are the associated uncivil behaviours? (2) What are the subsequent consequences of racially minoritised hospital workers experience of incivility? (3) What are the recommendations and research gaps related to incivilities experienced by racially minoritised hospital workers?

### Stage 2: Identifying relevant studies

The systematic search was conducted by the review team members, in collaboration with a lay leader^2^ and a university research librarian to locate original published and grey literature sources. A search strategy was developed using an iterative approach to modify search terms according to the Population, Concept, Context framework with synonyms for the population (racially minoritised hospital workers), concept (incivility) and context (hospital setting). The strategy also included eligibility criteria, identified databases and specified time spans. For further details, see Supporting Information S1. We identified relevant international literature between January 1999 to May 2021 through three main routes: (1) searches in eight research databases (MEDLINE, CINAHL, Cochrane Database of Systematic Reviews, Embase, Global Health, PsycINFO, Scopus and WorldCat) for published articles using search terms, (2) citation searching and hand‐searching and (3) international agencies and grey literature databases, such as World Health Organization, Global Health and Google. The primary search was conducted in August 2021.

### Stage 3: Selecting relevant studies

Two levels of screening were implemented for both academic and grey literature using the following approach: (1) title and abstract screening was conducted by a primary reviewer (OJ) based on the eligibility criteria. 10% of articles were split between two independent screeners (RL, BF); (2) the primary reviewer screened the full text of chosen articles, and the final list of articles was reviewed by (RL, BF and GM). The strength of agreement between reviewers at each stage of the screening process was discussed with the review team and disagreements were resolved.

**FIGURE 1:**
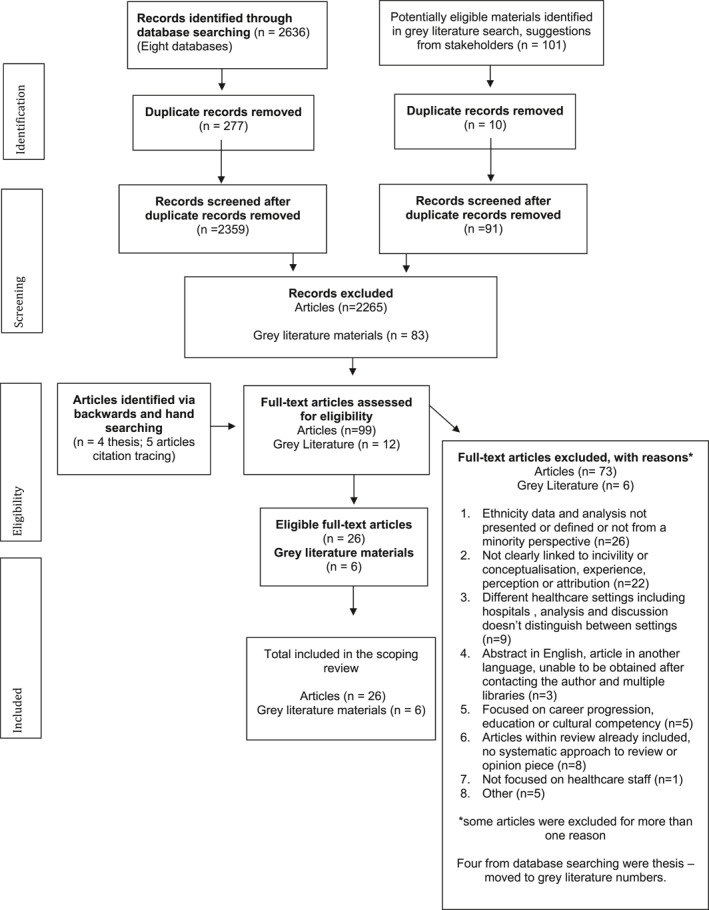
PRISMA flow diagram. PRISMA, preferred reporting items for systematic reviews and meta‐analyses.

A total of 2359 academic articles and 91 items of grey literature (including theses) were obtained after removal of duplicates. Following title and abstract screening with the inclusion criteria, 99 academic articles and 12 grey literature materials were moved into the full text screening phase. Twenty‐six academic articles and six grey literature materials (dissertations) were eligible for inclusion in the review. A descriptive summary of the key findings is presented following the PRISMA‐ScR guidelines (Tricco et al., 2018) (see Figure 1).

### Stage 4: Extracting the data

A total of 32 articles (studies and grey literature) fulfiled the inclusion criteria. Relevant data were extracted from included articles according to specific topics that addressed the research questions. The topic areas included study identifiers, for example, authors, year, country and population characteristics, for example, racially minoritised population, sample size, job role, speciality and concept of interest, for example, incivility, bullying, harassment, discrimination and context, for example, hospital type, department, ward, key findings for uncivil behaviours, experience and consequences (e.g. individual, work group, organisational and patient care processes). This is not an exhaustive list and further data charting categories can be found in Supporting Information S1. The primary reviewer (OJ) extracted the data, with subsequent verification by (RL, BF), and any disagreements were discussed and resolved by a third reviewer (GM), as required.

### Stage 5: Collating, summarising and reporting the results

In accordance with ethical guidelines for conducting a scientific literature review, a clear comprehensive auditing of the data collating, summarising and reporting process was undertaken. A narrative synthesis was undertaken utilising descriptive statistics, content analysis and thematic analysis dependent on the research questions (Popay, 2006). The most relevant analytical tools and techniques were chosen to develop a descriptive account of the best available evidence that addresses the research question. To characterise and explore each included article, we used descriptive statistics, for example, frequency counts with percentages to provide an overview of the body of included papers. We generated quantitative summaries of important elements of the articles, such as the study characteristics, and frequency of topics related to the research questions. To organise the data related to uncivil behaviours, consequences and recommendations, we conducted a basic qualitative content analysis with an inductive extraction and analysis and the coding framework was developed (Popay, 2006; Stemler, 2001) in collaboration with stakeholders (Popay, 2006). The recommendations were derived from studies that directly linked the recommendation's goal to elements connected to incivility; they were evaluated by the review panel to reject any suggestions that clearly address higher intensity phenomena and obtain consensus. For the large quantities of qualitative data related to the review question concerning incivility experiences, thematic analysis was used to identify and amalgamate key themes within and across articles with an inductive coding approach. For a detailed example, see Supporting Information S2.

### Stage 6: Consultation with relevant stakeholders

Similar to Daudt et al. (2013), this scoping review extended beyond one researcher, enhanced by involving multiple partners, including an interdisciplinary review team as well as racially minoritised staff, interested patients and members of the public throughout the research process. The staff, patients and members of the public provided critique and suggestions for the research questions, search strategy, eligibility criteria, data extraction form, sense checking data categorisation and interpretation during analysis and took part in interactive workshops to develop a flyer and visual findings to disseminate key research findings (see Supporting Informations S3 and S4).

### Quality appraisal

A critical appraisal of quality and risk of bias within included studies was not examined within this scoping study. Whilst there has been debate regarding this, a scoping review aims to map the extent of available knowledge on a given topic not to derive synthesised evidence for clinical use (Peters et al., 2020), thus a quality appraisal was not required.

## RESULTS

A summary of the characteristics and key review findings of included studies can be found in Supporting Information S5.

### Overview of included studies

**FIGURE 2:**
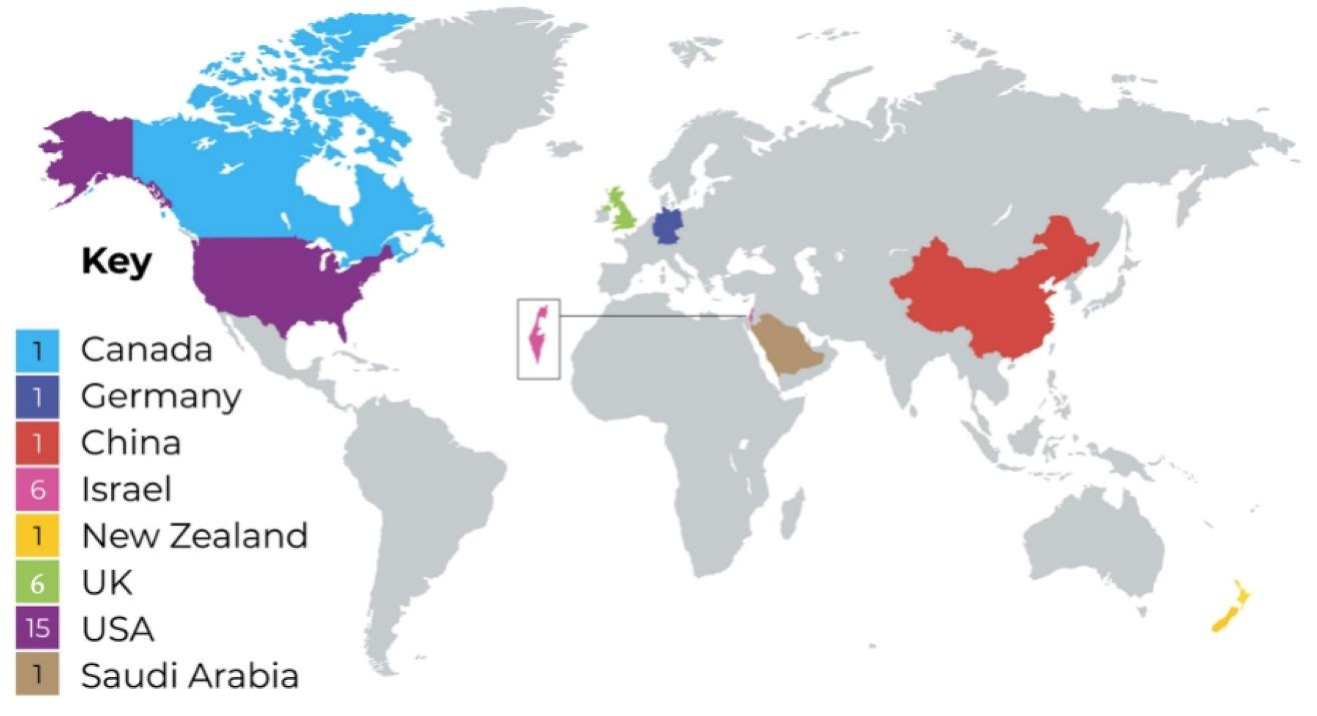
Map of countries of included studies.

Of the 32 included studies, 24 (72%) were published between 2016 and 2021. The sizes of racially minoritised populations ranged between 2 and 509 participants. Almost all the studies included nursing populations (*n* = 31, 97%). The studies were conducted in the following countries: the United States (*n* = 15), the United Kingdom (*n* = 6), Israel (*n* = 6), China (*n* = 1), Germany (*n* = 1), Canada (*n* = 1), New Zealand (*n* = 1) and Saudi Arabia (*n* = 1) (see Figure 2). Qualitative methodologies were the most common research methodology and semi‐structured interviews were the primary choice of method. Only four articles explicitly focused on exploring incivility within racially minoritised populations (Supporting Information S5: 1, 11, 30, 32), whereas the others focused on broader behavioural constructs, such as bullying, discrimination or harassment. However, uncivil behaviours and experiences were identified in the remaining 28 studies, using the original definition of incivility (Andersson & Pearson, 1999). The racial and/or ethnic composition of the study population defined by the authors included Black or African American (30.3%, *n* = 10), Asian American (6%, *n* = 2), Native American (6%, *n* = 2), Asian or Pacific Islander (9%, *n* = 3), Maori (3%, *n* = 1), Asian (9%, *n* = 3), Arab (18%, *n* = 6), Turkish background (3%, *n* = 1), and Hispanic or Latino (15%, *n* = 5). Broader grouped terms, such as ethnic minority, minority ethnic and multiple ethnicities (19%, *n* = 6) were primarily used in the United Kingdom. In summary, the body of evidence is largely qualitative with a limited range of participants from a small number of countries that include diversely defined study populations encompassing racial, ethnic, geographical and regional descriptions.

### Objective 1: Uncivil behaviours and experiences of incivility

#### Types of uncivil behaviours

Eight common types of uncivil behaviour were identified within incivility experiences from 26 articles, which were mainly qualitative (see Supporting Information S2). These behaviours include: (1) Questioning competence, authority and knowledge, (2) Unequal allocation of work tasks, patients, leave and training, (3) Verbal, non‐verbal and para‐verbal hostile behaviours, (4) Ignoring and dismissing, (5) Lack of support and help, (6) Indirect care or treatment refusal, (7) Ascribed or perceived stereotypes, insensitivity and identity erasure, 8. Heightened scrutiny and criticism.

The three most frequently described behaviours were categorised as hostile through verbal, non‐verbal and para‐verbal expressions, imposition of stereotypes, insensitivity and identity erasure, and unequal allocation of work tasks, patients, leave and training. Further detail is provided in Table 1.

**TABLE 1:** Example of the top three categories of uncivil behaviours experienced by racially minoritised hospital workers.

Type of uncivil behaviour	Subtypes of uncivil behaviours	Examples of behaviour (with references [Supporting Information S5] in brackets)
Verbal, non‐verbal and para‐verbal hostile behaviours	Gossiping	Gossiping (6, 11, 28)
	Hostile behaviours	Hostile behaviours (29, 30)
		Argumentative (20)
		Interruptions during discussions, meetings and general conversations (5, 28)
		Walk away during communication (28)
		Unfriendliness (15, 30)
		Cursing (16)
	Rudeness	Rude remarks (6)
		Derogatory remarks (8)
		Rudeness from peers and colleagues (28, 29)
	Lack of respect/disrespectful behaviours	Lack of respect—Colleagues (4, 11, 18)
		Lack of respect—Manager (26)
	Yelling	Screaming (20)
		Yelled at (31)
	Talking in rough and harsh tones	Harsh words (8, 31)
		Talking ‘rough’ (31)
		Berated—Criticised angrily (31)
	Hostile looks	Accusatory looks (8, 18)
		Hostile looks/glances (14)
	Snide comments	Sarcasm (4)
		Snide comments (6)
		Insensitive comments (6)
		Defensive remarks (14)
	Negative remarks	Negative remarks (4, 19)
		Insulting behaviours (21)
		Negative comments from patients and families (25)
		Negative comments from colleagues (4, 19, 25)
Ascribed or perceived stereotypes, insensitivity and identity erasure	Stereotyping—Asking personal or professional questions	Stereotyping/assumptions based on surface identity (5, 6, 12)
		Asked to explain patients culture to peers (19)
		Assuming job role based on stereotypes—Patients and physicians (7, 31)
		Questioning position, title, degree level (11)
	Inappropriate joke—Mocking factors related to identity for example, accent	Racialised colloquialisms (5)
		Inappropriate jokes (23)
		Making fun of someone (26)
		Mockery of accent or remarks about nationality (26, 30)
	Expressing negative views related to racialised or cultural group	Expressing political views about staff racial group (8)
		Stereotypical remarks about patients with same racialised group as staff (11, 15 19, 25)
		Inappropriate comments regarding terror attacks or war (16)
		Disagreements related to identity (20)
	Cultural assumptions and insensitivity and ignorance	Culturally ignorant remarks and assumptions—Staff (6, 10, 11, 20)
		Religious insensitive remarks—Staff (6)
		Personal comments about religious practices (6)
		Inappropriate, insensitive cultural questions—Patient (6)
		Lack of culturally appropriate work social activities (28)
	Changing name without permission for example, nicknames	Nickname given without permission (28)
		Not addressed by name (28)
		Not learning their names (28)
Unequal allocation of work tasks, patients, leave and training	Unreasonable refusal of leave, training and promotions	Unfair treatment in requests for training (3)
		Unreasonable refusal of leave, training and promotions (23)
	Unequal work scheduling	Unequal work scheduling and flexibility than majority peers (28, 32)
		Unequal work flexibility than majority peers (28)
	Unequal workload	Lack of support from colleagues (not relieved for breaks) (4)
		Unequal work allocations than majority peers (18, 28)
	Unequal work assignments	Moved to different departments in the hospital (3)
		Unfair work allocations than majority peers for example, harder patients (10, 11, 25)
		Unfair work allocation to translate for patients or allocated patients of similar racialised group (19)

#### Experiences of incivility

**FIGURE 3:**
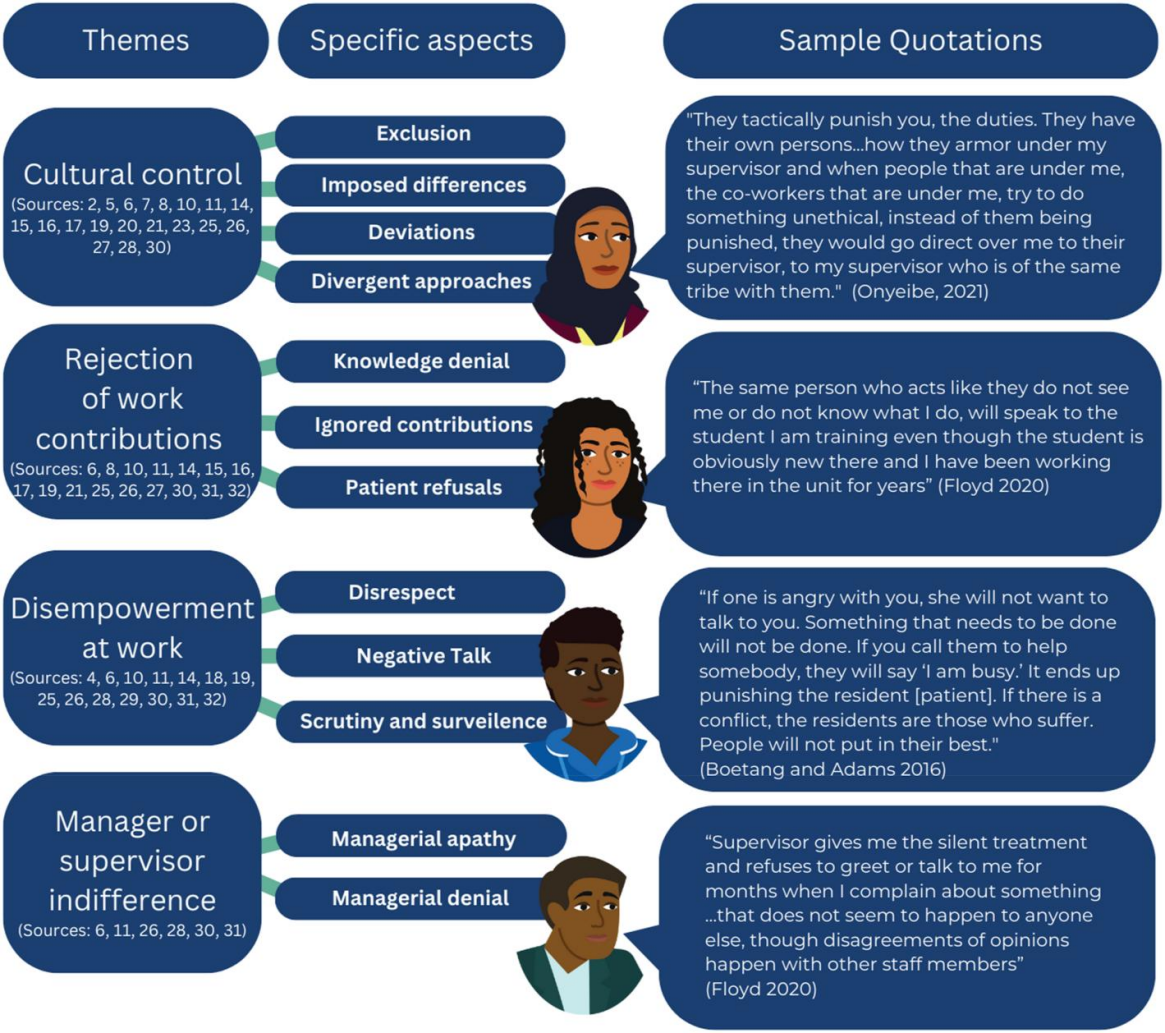
Example of racially minoritised hospital workers experiences of incivility.

Across 25 of the included studies, study participants shared a range of behaviours that often overlapped and converged to violate mutual respect according to the original definition. This review generated four major themes related to how racially minoritised workers experience incivility: (1) cultural control, (2) rejection of work contributions, (3) disempowerment at work and (4) manager or supervisor indifference or lack of concern about incivility. An example of the specific aspects and quotations related to each of the themes is outlined in Figure 3.

#### Cultural control

Racially minoritised workers experience incivility through social and behavioural exclusionary processes that reinforce who is included in work and non‐work related interactions. Nine studies (Supporting Information S5: 5, 6, 7, 10, 11, 17, 26, 28, 30) outlined the experiences of racially minoritised staff navigating social groups of colleagues within the workplace. Cliques^3^ were described as facilitating exclusion from work discussions that took place in social spaces (Supporting Information S5: 7, 10), influencing work assignments and tasks (Supporting Information S5: 5, 6, 11, 28, 30), as well as supporting the tactical avoidance of the authority or knowledge of Black nurses (Supporting Information S5: 7, 10, 11, 17). These were mostly experienced by Black and African nurses and nurse associates in the US.

Several articles report the use of negative signals expressed through direct actions such as not learning or using a person’s name (Supporting Information S5: 26, 28), negative reactions to hearing minoritised languages (Supporting Information S5: 20) or indirect actions (Supporting Information S5: 8, 10, 15, 23), such as the absence of certain staff from a team list on a patient board (Supporting Information S5: 10). These experiences suggest differential social dynamics and team interactions for racially minoritised staff that signal that they are on the periphery of the workgroup, which reinforces messaging that they are not valued by their team.

Fifteen articles (Supporting Information S5: 2, 5, 6, 8, 10, 11, 15, 17, 19, 21, 25, 26, 27, 28, 30) reported several ways colleagues impose differences to racially minoritised hospital staff, highlighting colleagues imagined cultural demarcations and knowingly or unknowingly emphasising cultural differences underpinned by prejudice and stereotypes in the workplace. These impositions related to identity included intrusive personal and professional remarks and questions, raising awareness of physical characteristics (e.g. hair), assigning patients related to an assumed similar identity, or witnessing derogatory remarks about people with a similar or the same identity (Supporting Information S5: 6, 8, 11, 15, 19, 21, 25, 26). Two articles (Supporting Information S5: 6 and 27) examined the work experiences of Jewish nurses and Turkish or Turkish heritage German inpatient geriatric staff, respectively, and reported pejorative comments directed towards religious minorities. The interview studies found that colleagues would ask inappropriate questions about their religion and expect staff to answer questions on behalf of a perceived homogenous negatively racialised or religious group.

Further to this, two articles outlined negative encounters when organising work requirements (Supporting Information S5: 6, 14). Colleagues would question, criticise and respond negatively to any deviations from their expectations within the workplace. One interview study of Jewish nurses’ work experiences in a New York hospital reported several accounts of colleagues’ resistance to deviations from the ‘usual’ practices in the workplace (Supporting Information S5: 6). The nurses experienced uncivil interactions whilst scheduling work around religious holiday despite following the appropriate work processes (Supporting Information S5: 6). The manager’s expectations for work flexibility with staff were met with hostile remarks, assumptions and misunderstandings. For example, head coverings such as wigs and headscarves (tichel) and scrub dresses in a labour and delivery ward were met with questioning or commentary from peers, despite the nurses being granted permission to wear them (Supporting Information S5: 6).

Another related but distinctive constructed subtheme was uncivil work experiences between colleagues related to divergent work approaches and communication styles. One interview study in New Zealand found that internationally educated nurses reported that host (majority) colleagues expressed frustration and criticism towards their checking and double‐checking practices (Supporting Information S5: 5). In addition, a Samoan nurse shared their experience of trying to integrate into a work culture that normalised uncivil behaviours, with colleagues interrupting during meetings and conversations (Supporting Information S5: 5). Similarly, a thesis exploring native African nurses’ experiences of work environments in the US found that a male Nigerian nurse experienced accusations of yelling at a colleague when he first started at work, but described he was talking appropriately (Supporting Information S5: 30). He perceived the uncivil interaction to be a difference in the way people speak in the US compared to Nigeria. He describes this occurring at orientation, which suggests that incivility can occur even during integration processes within a workgroup. Interestingly, an interview study exploring nurses’ experiences at the bedside examining race, gender and emotions work found evidence of the racialisation of emotional labour with disproportionate work expectations for emotional management and performance expected for Black female nurses compared with staff racialised as White (Supporting Information S5: 7).

#### Rejection of work contributions

Incivility as rejection refers to the myriad of ways that racially minoritised workers report having their expertise, knowledge, suggestions, contributions and practical caring work dismissed or denied from multiple sources such as patients, relatives, colleagues and managers. Four articles found that Black nurses, operating room technicians and patient care technicians experienced negative assumptions regarding their expertise and skills, experienced through a lack of acknowledgement (Supporting Information S5: 6, 10, 11, 17).

Similarly, other articles shared respondents' experiences of not being seen despite their efforts to contribute suggestions, offer training or occupying a role in authority (Supporting Information S5: 17, 25, 26). Findings from three interview studies describe African Born Black, African American and native African minority nurses experiences of being ignored when asking for support from colleagues to make decisions about patient care or share care information (Supporting Information S5: 28, 30, 31).

Interestingly, rejections by patient or patient relatives via covert body language, expressions and the guise of preference were reported in three articles within the context of Germany (Supporting Information S5: 27) and the US (Supporting Information S5: 21, 30). Findings stated by Turkish heritage nurses, Arab and Black African American nurses include being ignored, making signals indicating a desire not to be touched and doing the opposite behaviour requested of them. The Black African American nurse with an accent described rehearsed responses from patients that suggested the patient did not know what they were saying (Supporting Information S5: 21). It is worth noting that several studies also reported explicit care refusals from patients or family members (Supporting Information S5: 6, 8, 10, 15, 19, 21, 25, 32). One study in Israel specifically examined treatment refusal experienced by Arab nurses and doctors; treatment refusals were mainly overt and there was only one indirect care refusal experience where an Arab nurse trying to get a patient to change position in bed was asked not to care for the patient by a Jewish family member.

Four studies examined workplace experiences within the workgroup and between staff and patients, considering workgroup diversity (Supporting Information S5: 8, 14, 15, 16). Each of the studies considered interpersonal relationships between staff in the context of the ongoing Israeli colonisation of Palestinian land, and heightened tensions in response to proximity to hostile events. These studies report increased negative emotions from patients and relatives within hospital environments, such as fear, anxiety, worry expressed through accusatory looks, body language, cursing (Supporting Information S5: 14, 15, 16) and inappropriate language (Supporting Information S5: 8). Similarly, studies in the US and Germany, exploring experience of Jewish (Supporting Information S5: 6), Hispanic (Supporting Information S5: 25) and Turkish Heritage nurses (Supporting Information S5: 27) report patients making insensitive comments about their identity without realising they belong to that group, particularly not accepting that they belong to the group such as ‘you don’t look like an Arab’.

#### Disempowerment at work

Racially minoritised nurses and operating room technicians, mainly in the US (Supporting Information S5: 4, 18, 19, 28, 30) and Canada (Supporting Information S5: 32), reported incidences of their needs not being met or respected by colleagues through a lack of help and support during care processes. These experiences include a lack of relief or support to take a break (Supporting Information S5: 4, 18, 28), colleagues not relaying appropriate information (Supporting Information S5: 28) and ignoring when questioned and purposeful unhelpfulness in response to previous conflict (Supporting Information S5: 30, 32). The inaction of team members reduced the control and autonomy that racially minoritised workers (primarily Black, Latin(o) and visible minorities) had over their care work.

Another avenue for disempowerment identified in the literature involved negative talk such as hostile and disrespectful behaviour experienced from co‐workers and supervisors or those in positions of authority such as preceptors (Supporting Information S5: 18, 26, 28, 30). Minority, Black and African Born (primarily female) nurses specifically report experiences involving doctors berating, using harsh words, yelling and talking down to them when asking for support with a patient (Supporting Information S5: 11, 26, 28, 31) or providing quality and safety related training (Supporting Information S5: 11). Feelings of not being listened to and being ignored were commonly described. In contrast to being ignored, four qualitative studies highlighted minority nurses’ experiences of indirect negative talk through being the focus of colleagues gossiping about their work ethics (Supporting Information S5: 11), sharing essential information behind ones back (Supporting Information S5: 28), witnessing management discuss other colleagues (Supporting Information S5: 26) and false accusations (Supporting Information S5: 6, 26). Additionally, two thesis’s provided evidence of the lack of respect and absence of kindness expressed towards African American nurses and operating technicians from physicians through rude remarks, yelling and how they are treated that was not observed towards African American male nurses (Supporting Information S5: 4, 31). These findings point to a complicated interaction between raced and gendered identities within experiences of incivility.

Heightened scrutiny and surveillance from colleagues and management were common experiences of incivility described by Hispanic, African American and minority nurses (Supporting Information S5: 11, 25, 30, 31). The forms of scrutiny included colleagues following nurses to check up on them (Supporting Information S5: 11), nitpicking (Supporting Information S5: 32), colleagues anticipating mistakes and differentially addressing them (Supporting Information S5: 25, 30, 31). One author describes these experiences as being ‘negatively visible’ (Supporting Information S5: 25). Four interview studies (Supporting Information S5: 10, 11, 18, 31) and one survey (Supporting Information S5: 25) conducted in the US provided examples of support associates and nurses witnessing or receiving higher and harder patient assignments than their White majority colleagues.

#### Manager or supervisor indifference or lack of concern about incivility

Six US studies provided detailed accounts of nurses’ experiences of apathy related to raising concerns related to racial bias, uncivil interactions from colleagues and sharing apprehensions or ideas about changes at work (Supporting Information S5: 6, 11, 26, 28, 30, 31). Two studies suggest that doctors are held in higher regard than the nurses and their side of an incident was believed or dismissed as ‘normal’ behaviour (Supporting Information S5: 26, 28) and one qualitative study described retaliatory silent treatment from a supervisor that lasted for months in response to a minority nurse putting in a complaint (Supporting Information S5: 11).

### Objective 2: Consequences of incivility experiences

Approximately 66% (*n* = 22) of all included articles reported negative impacts of incivility. Consequences were identified across three major categories outlined. First, the individual hospital workers' psychological and physiological health and wellbeing. Second, the workgroup dynamics and associated patient care processes. Finally, threats to the organisation.

Negative emotions and feelings were the most identified consequence for the individual target of incivility, accounting for 54.4% (*n* = 18) of included articles. Of the 22 negative emotions, the main emotions were frustration (Supporting Information S5: 6, 7, 26, 30, 31) and anger (Supporting Information S5: 7, 15, 21), whilst the key feelings were insult (Supporting Information S5: 11, 21) and humiliation (Supporting Information S5: 11, 15, 21).

Workgroup relations were harmed by uncivil encounters in 30% (*n* = 10) of the papers, with 15 reported reductions in support and help‐seeking behaviours (Supporting Information S5: 5, 10, 11, 14, 15, 18, 19, 20, 28, 32). The lack of collegial support was demonstrated through unhelpful behaviours (Supporting Information S5: 18, 19, 28, 32). Consequences related to patient care delivery were sparsely present within 9% (*n* = 3) of articles. Only three articles referenced compromised patient care processes, including poorer communication with patients via being short with a patient (Supporting Information S5: 6), not listening to patients appropriately (Supporting Information S5: 31) and reduced care due to the lack of timely support from colleagues resulting in the decline of responsiveness to patient needs (Supporting Information S5: 32). Consequences that jeopardise hospital organisations were found in 27% (*n* = 9) of the articles, with 25 distinct descriptions of organisational risks. The hospital organisations faced the most significant risks in terms of employee retention, primarily stemming from feelings of not belonging and experiencing isolation (Supporting Information S5: 5, 6, 10, 15, 25, 28, 32), as well as a diminished sense of being able to bring their authentic selves to work (Supporting Information S5: 5, 6, 8, 15, 20). These factors, in turn, were identified as disruptors of social cohesion within the workforce, as they compromised employees' sense of belonging and posed a threat to workforce retention by heightening the desire or intent to leave.

### Objective 3: Common interventions, improvement initiatives, research gaps and recommendations to address incivility

Based on the knowledge obtained from participants, the study authors presented informed suggestions about how to address uncivil behaviours and experiences. Five areas of recommendation with multifaceted approaches were categorised, which focused on the following areas: (1) Creating inclusive environments, (2) Developing evidence‐based intervention programmes, (3) Cultivating proactive structural competency, (4) Adapting to the work environment and (5) Supporting effective leadership.

#### Creating inclusive environments

Eight articles (primarily qualitative designs using interviews, group interview and open‐ended survey questions) addressed the need to improve cultural sensitivity and competence within teams (Supporting Information S5: 5, 6, 11, 18, 19, 25, 27, 28). Four of these suggested that training for workers to address their own biases and recognise the biases of others would improve cultural sensitivity and understanding between colleagues in diverse teams (Supporting Information S5: 5, 6, 11, 18, 27, 28). Three studies recommended extending beyond individuals to addressing biases within the work climate through leader learning and confronting biased behaviours or language about marginalised staff and patients (Supporting Information S5: 19, 25, 28). Finally, six articles identified the importance of clear, fair and trustworthy reporting mechanisms (Supporting Information S5: 12, 17, 19, 25, 26, 29) with multiple avenues to report, including anonymous reporting mechanisms (Supporting Information S5: 26).

#### Developing evidence‐based intervention programmes

Common intervention programmes to address incivilities included individual and group training focused on developing communication skills (Supporting Information S5: 1, 2, 5, 6, 17, 19, 25, 27), assertiveness (Supporting Information S5: 2, 13), resilience (Supporting Information S5: 6, 19), stress and conflict management (Supporting Information S5: 1, 18, 32). Seven articles recommended investing in mentoring programmes as an intervention to improve support primarily for nurses (Supporting Information S5: 5, 6, 11, 18, 19, 25) and other hospital workers (Supporting Information S5: 22). These were suggested to address othering, improve openness and understanding of differences and improve resilience and retention. Three articles emphasised the importance of training leaders to be responsible for encouraging workgroup inclusion by addressing their own biases and confidently identifying and addressing ‘unintentional and unconscious’ behaviours (Supporting Information S5: 19, 10, 28). At an organisational level, the US Department of Veterans Affairs developed the Civility, Respect and Engagement Workforce intervention. This aimed to improve workgroup civility through a group level tailored intervention with pre and post civility assessments. It involved multidisciplinary commitment to regular facilitated meetings over 6 months with shared objectives, expectations, and cognitive rehearsal focusing on what to do when experiencing incivility. The results showed only a marginal increase in civility that remained consistent a year later; however, despite evidence that this intervention represented the most effective approach, further strategies or adjustments are required to achieve a more significant improvement (Supporting Information S5: 13).

#### Cultivating proactive structural competency

In this context, structural competency refers to organisational and health‐care worker awareness and ability to recognise and respond to the influence of social structures on interactions and the practice of health care (Downey & Thompson‐Lastad, 2023; Metzl & Hansen, 2014). The structural aspect moves beyond individual responsibilities to recognise the organisational policies, practices and governance and wider social, political and economic factors that can shape uncivil interpersonal interactions (Metzl & Hansen, 2014). Several articles report that it is essential to be proactive in organisational policies, training and work approaches to address incivilities and similar workplace aggression experienced by racially minoritised employees from colleagues, management and patients (Supporting Information S5: 20, 21, 22, 26, 28, 29, 30, 31).

#### Adapting the physical work environment

Two articles proposed physical adaptations to the work environment for two separate reasons (Supporting Information S5: 2, 31). A survey study suggested quiet spaces and relaxation or renewal rooms to provide a physical space for de‐escalation and stress reduction to reduce disruptive behaviours (Supporting Information S5: 2).

#### Supporting effective leadership

Recommendations for the role of leadership in addressing uncivil behaviours primarily focused on improving the supportive behaviours of nurses’ and physicians in leadership positions (Supporting Information S5: 6, 11, 15, 19, 20, 28, 30, 32). The varied suggestions included regular supervisory check ins (Supporting Information S5: 11), role modelling positive behaviours (Supporting Information S5: 2), acknowledging and rewarding the additional (often invisible) cultural brokerage work of minority employees (Supporting Information S5: 19) and continuous learning with interdisciplinary team members, particularly within discussions related to cultural sensitivity (Supporting Information S5: 19, 20).

Furthermore, leadership's role in fostering a supportive environment with institutional support for positive intergroup contact (Supporting Information S5: 15, 28), promoting cross‐cultural understanding at all levels and addressing inappropriate conduct effectively were considered to contribute to better communication and work experiences (Supporting Information S5: 19, 28). Lastly, valuing and encouraging authentic expression and belonging for everyone including ethnic minorities was recommended to improve work group unity (Supporting Information S5: 20, 28, 32).

## DISCUSSION

This is, to the best of the authors' knowledge, the first international scoping review to identify and demonstrate the extent of available evidence regarding incivility experiences of racially minoritised hospital workers including uncivil behaviours and consequences. This narrative synthesis shows how incivilities experienced by racially minoritised hospital workers manifest through work duties and processes and casual social interactions. A substantial amount of evidence documented the negative impact of incivility on the victim; however, some studies highlighted the negative implications of incivility on workgroup relations, performance, and patient care processes.

The most common example of uncivil behaviours involved hostile behaviours, ignorance, insensitivity and erasure and unequal allocation of work tasks, patients, leave and training. These behaviours are expressed during interpersonal communication, which is an essential component for developing rapport, trust and effective relationships within and between hospital teams, patients and visitors. Further, these important factors facilitate safe care performance within hospital teams (Baxter et al., 2019). The quality of communication plays a vital role in maintaining wellbeing, patient safety and quality of care due to care coordination activities, for example, receiving and relaying information, listening to patient concerns and needs (Iedema et al., 2019). Staff‐to‐staff and patient‐to‐staff communication involves verbal (e.g. oral, audible or written), non‐verbal (e.g. body language and expression) and para‐verbal (e.g. tone, pitch, pace, level of voice and intonation) intentional and unintentional signals, which can be negatively or positively received (Iedema et al., 2019). Uncivil behaviours span across both passive and active forms, aligning with Floyd's (2020) proposed racialised spectrum of incivility and bullying. Racially minoritised workers predominantly experience passive incivility, such as being ignored, dismissed, disregarded, and gossiped about in various work duties and casual social situations. Passive incivility, marked by inaction and subtle disrespect, has a significant impact, as shown in empirical research (Abate & Greenberg, 2023; Cortina et al., 2011; Credland & Whitfield, 2021). Conversely, active expressions, such as berating, scrutinising and intrusive commentary, are more overt. Hamed et al.'s (2022) global scoping review on racism in health care reveals a lack of consistent conceptualisations of racism, emphasising that racism is often normalised and hidden behind ostensibly non‐racial practices in healthcare. This underscores the embedded nature of racial dynamics in organisational structures (Ray, 2019). It is crucial to recognise the diversity and subtlety of these behaviours, noting the interplay between passive and active displays of incivility.

Experiences of incivility were described through processes of othering, rejection, disempowerment and apathy from management. Racially minoritised workers (primarily nurses) experienced adjustments or contention between personal cultural norms related to identity and workplace norms concerning communication and work styles. Collectively these experiences represented a form of norm policing whereby colleagues and managers that primarily benefit from or are used to specific norms become defenders or enforcers of the boundaries of these norms and the behaviours or approaches to be integrated or upheld. Notably, Abate and Greenberg (2023) reviewed incivility in North American medical education and found medical students were exposed to different types of incivility including gendered and racialised experiences during training placements. They highlighted the influence of power within health‐care systems coupled with ingrained negative behavioural norms contribute to the early adoption and normalisation of incivility. These findings are corroborated in the literature on discourses about power in hierarchal, racialised organisations (Ray, 2019, Woodhead et al., 2021). Building on the work of Foucault and Weber, Collins outlines four aspects of power demonstration, which includes structural, disciplinary, interpersonal and hegemonic (Alinia, 2015; Collins, 2009). The latter two aspects can help to explain how hegemonic processes (maintaining the beliefs, values and ideas of the dominant group through language and imagery) interact with in‐group outgroup processes to legitimise othering. These processes also work alongside the interpersonal domain that influences how an individual sees themselves and their experiences in relation to others and may in fact support suppression of individuality and authenticity through subtle corrective uncivil actions.

While incivility has been shown to operate at the individual level, evidence within included studies shows that uncivil behaviour can be reinforced by organisational factors, such as unclear implementation strategies for zero tolerance policies, manager passivity or apathy towards unacceptable behaviour and management or organisation of work (e.g. staffing levels, workloads). This corroborates discussions in the wider literature that incivility can operate and requires analysis at a micro (interpersonal), meso (organisational) and macro (societal) level (Mir, 2020; Ozturk & Berber, 2022). Cortina (2008) draws on social identity theory and categorisation theory to emphasise the discriminatory underpinnings of incivility, which can be subtle, covert forms of racial prejudice and discrimination. The perception of uncivil behaviour or context, as well as the logic given to why it occurred, can affect the evaluation, subsequent responses and consequences of incivility (Schilpzand et al., 2014). This can be influenced by the ambiguity of intent, which is fundamental to the characteristics of incivility, and subjective appraisal can alter whether a target is offended or not (Bunk & Magley, 2013; Kern & Grandey, 2009), supporting the assertion that differences in incivility experiences and consequences among racially minoritised employees in healthcare warrants further investigation. Furthermore, organisational norms and expectations for how emotions are expressed, regulated and managed are communicated formally and informally and subject to both racialised and gendered dynamics. Wingfield (2010) demonstrated how feeling rules are influenced by gendered social stereotypes, which are often intertwined with racialisation. A recent review found that racially minoritised women (specifically racialised as Black) undertake additional emotional labour to deal with the racial undertones of interactions with colleagues and service users that perceive expertise, intellect and ability in relation to racialised identity (Wingfield, 2021, p. 201). Historically rooted pervasive stereotypes can underpin pervasive actions and shape perceptions of uncivil interactions and attribution of emotion to disposition (e.g. personality) rather than situational (e.g. environment, context) (Motro et al.^2022^; Wingfield, 2021). Additionally, there is a reluctance to display certain emotions and complex work to control emotional expressions (e.g. anger, frustration) due to understanding of racialised negative stereotypes, assumptions and tropes linked to their social identities such as the ‘Angry Black Woman’ trope (Motro et al., 2022). The findings by Floyd (2020), Cottingham et al. (2017), Pierce (2018) and Brooks (2016) show that nurses from minoritised ‘race’ and gender groups face heightened racialised burdens. These burdens occur when work situations require an emotional display that does not reflect how they really feel in the face of racialised and gendered stereotypes during care processes and interactions with colleagues. Further, a narrative review of patient incivility towards healthcare providers by Townsley et al. (2023) stipulates the lack of significant evidence on predicted characteristics for the risk of patient incivility. The review found that nurses who use emotion work to negotiate incivility experience more fatigue compared to those responding authentically. Whilst these findings, only involve nursing professions, where ‘race’ and gender are most likely to intersect, they also highlight the importance of understanding the role of emotional climate and how and in what ways feelings, attitudes and emotional expectations are disproportionately managed across the workforce.

The consequences of incivility are varied and operate at multiple levels, individual, interpersonal (workgroup and patients) and organisational. At an individual level, negative emotions and feelings were prominent consequences of incivility. There was a combination of visceral immediate affective responses (e.g. fear, shock, sadness and anger), short term affective responses when cognitive processing starts to occur (e.g. insulted, humiliated, hurt and disheartened) and longer‐term reduction of mental health (e.g. anxiety, burn‐out and hypervigilance). This complements previous empirical evidence described by Heyhoe (2013), which outlined three types of emotion, including mood states (positive or negative enduring emotions), anticipatory affect (immediate strong reaction to a stimulus often based on past events), anticipated affect (thoughts of future feelings in relation to others) and the association with negative safety outcomes via poorer decision‐making processes. This review adds to the growing calls to conduct more research to deepen our understanding about how emotion impacts patient safety (Heyhoe et al., 2015; Heyhoe & Lawton, 2020). Further research could usefully explore the relationship between affect and incivility, with affective responses as a trigger of incivility and an outcome, that can occur through spiral processes outlined by Andersson and Pearson (1999) as an ‘incivility spiral’. They proposed that an uncivil act could lead to reciprocated acts that can escalate into higher severity behaviours. Incivility may be a stimulus for disrupting the emotional state of individuals and workgroups via social emotion processes and subsequently lead to negative implications for work communication and care processes. It is important to note that in the review, there were no studies that specifically examined safety outcomes with direct patient implications in association with incivility experiences, such as patient falls, adverse events, near misses, risks, increased infection, errors/error rates and complication rates. However, many of the individual and workgroup consequences, such as cognitive depletion (Riskin et al., 2019), reduced individual and team performance (Hicks & Stavropoulou, 2022; Katz et al., 2019) and employee silence (Katz et al., 2019; Salazar et al., 2014) have been linked to reductions in the quality and safety of patient care. It is worth noting that these negative consequences have the potential to endanger the capabilities or capacity of staff to identify hazards and risks to patients, essentially impairing safety preparedness and responsiveness and increasing the likelihood of avoidable harm to patients (Guo et al., 2022; Riskin et al., 2019). Thus, the relationship between incivility, employee silence and an employee's ability to voice concerns, make errors and share opinions (namely psychological safety, Edmondson, 1999), as well as the influence of racialised disparities, requires additional examination. Furthermore, our analysis reveals that incivility may have an impact on worker turnover and staffing instability, both of which can be costly. In addition to limiting the contributions, recommendations and possibilities for career development for staff from racially minoritised backgrounds, these factors may be a significant motivator to focus on eliminating incivility as a potential low‐cost way to enhancing safety culture. These studies indicate that exposing racially minoritised hospital workers to incivilities has a wide range of personal, team and organisational implications.

The review contributes promising recommendations for interventions that reduce incivility within the workforce. Several studies called for the inclusion of diverse populations within incivility research, including varied personal and professional demographics (e.g. different minority identities and expanding beyond nursing and physician roles). Furthermore, gaps in present data fail to account for individuals holding multiple privileged and oppressed identities, and there is a need to integrate studies of intersectional identities, notably race and gender, that function at distinct sites of oppression or compound to generate unique experiences. In alignment with the multilevel consequences of incivility, existing research suggests that interventions to address negative outcomes should also be multifaceted focusing on individuals, workgroups and organisational factors. Our findings align with existing evidence that calls for multilevel strategies to address workforce inequity within healthcare systems (Salway et al., 2016). Several healthcare organisations including professional bodies and regulators emphasise the importance of adapting health‐care systems in response to rising population diversity to improve access, experience and outcomes of culturally and linguistically diverse patients. One such strategy involves enhancing cultural competence to address cultural and linguistic distance by reducing biases, improving curiosity and promoting respect (ICN, 2013; Kline & Somra, 2021; NMC, 2018; The Joint Commission, 2010). Notably, despite antidiscrimination efforts via designated equality, diversity and inclusion (EDI) roles and initiatives, changes in racial inequity have been minimal with the maintenance of a racialised hierarchy where power is concentrated in the ‘snowy white peaks’ of health‐care systems with racialised minorities absent in decision‐making positions (Kline, 2014). Government officials within the UK National Healthcare System have nevertheless, called for redirecting resources for EDI roles and consultation to external EDI firms, citing better uses of funds for recruitment of frontline staff (Rimmer, 2023). Yet, only 0.03% (£40 million) of the 23/24 budget allocated to EDI roles (Saddler, 2023). Officials have faced heavy, sector‐wide criticism from NHS leaders, which emphasised the moral, financial and business case for the essential allocation of EDI roles in terms of proactive commitment to legislative compliance, accountability to fair and inclusive practices and addressing the ongoing challenges of staffing recruitment and retention, which relies on international workers. Further, an analysis of NHS staff survey in 2022 highlighted that a lack of equality and diversity was the most prevalent driver of staff intention to leave (Tikhonovsky, 2023). These ongoing discourses about the importance of EDI resourcing threatens commitment to addressing workforce inequities.

This study strengthens calls for leaders to formally acknowledge the value and importance of knowledge translation and ‘cultural brokering’ that employed racially minoritised staff undertake and for this often hidden work to be embedded into rewarding processes, for example, appraisals, promotions and demonstrations of good practices (Moceri, 2012). Also, in concordance with existing literature, the review findings demonstrate that organisational listening to racially minoritised staff is ineffective and recommendations to embed robust processes for listening are congruent with wider literature and reports (Kline & Somra, 2021; Shepherd et al., 2019). However, there are concerns about organisational lack of regard for racially minoritised employees’ issues and a need to address barriers to them speaking up (Kline & Somra, 2021). Despite organisational initiatives and roles such as freedom to speak up in the UK NHS, there is a noted lack of knowledge regarding systemic cross‐cultural understanding of unequal power dynamics, social systems, knowledge of historical and contemporary systemic challenges and continuous reflexivity (awareness and reflection on one's own personal biases) (Shepherd et al., 2019). These findings strengthen the need for structural cultural competency in addition to the interpersonal cultural competency of healthcare professionals.

## IMPLICATIONS

Ultimately, the consequences of incivility suggest it is critical to implement the common multi‐level recommendations put forth in a variety of the included publications. For instance, the need to create inclusive environments within which cultural sensitivity, competence and recognition of biases across teams are embedded; the need to be proactive in implementing relevant organisational policies, practices and governance and the need to improve the supportive behaviours of those in leadership positions. Our review reveals a fragmented and disparate body of existing evidence concerning uncivil behaviours, experiences and consequences explored mainly through qualitative methodologies. We highlight the need for more research that can deepen our understanding of the experiences of racially minoritised experiences and negative outcomes related to incivility, to identify any moderating or mediating effects and potential barriers and facilitators. This will help to address experiences prior to escalation to higher intensity behaviours, such as bullying and discrimination, disciplinary processes or preventative harm to staff and patients. Our focus on safety and quality of care identified gaps in evidence of the association between the effect of negative emotion on information sharing and seeking, silencing and poorer perceptions of care quality. More empirical evidence of the influence of incivility on processes and mechanisms that are involved in the deterioration of the safety and quality of care provided for patients is needed.

## REVIEW STRENGTHS AND LIMITATIONS

### Strengths

A useful strength of our review was the inclusion of both quantitative and qualitative methodologies improved the breadth and inclusivity of studies to improve our understanding of the topic. Another strength of the review included the incorporation of secondary reviewers and consensus discussions at different stages throughout the screening, extraction and synthesis process, which increased the quality, reliability and ethical standards of the review. Lastly, the reviewers followed an evidence‐based systematic reporting process that clearly outlined the relevant steps needed to ensure quality (Tricco et al., 2018), including comprehensive engagement with racially minoritised staff at all levels, and patients or carers with an in this topic area. Sense‐making and discussion workshops strengthened our understanding of the phenomena and decision‐making, especially during prioritisation, analysis and dissemination.

### Limitations

A key limitation of this scoping review was the decision to include higher intensity concepts related to incivility, which may have reduced the specificity of the review. However, we were aware that lower intensity behaviours can co‐occur with higher intensity behaviours and the conceptual overlaps allowed us to critically appraise publications that used the definition of incivility to highlight behaviours that would be considered uncivil.

Despite our efforts to include international databases including WorldCat, the included studies were primarily conducted within a few countries in the Global North (US, UK, Canada, New Zealand and Germany) with far fewer in the Global South (China and South Africa). The geographical limitations were linked to the language limitation of English only inclusion criteria, which caused selection biases. We observed a substantial absence of publications, from the Global South in particular, in commonly searched databases, indicating a lack of relevant studies on incivility conducted in most countries and a need for more studies in diverse contexts. Despite our attempts to identify a wider range of staff roles, included studies predominantly involved nurses, which may affect the applicability of findings to lower paid, non‐professional health‐care support roles such as porters and cleaners.

## CONCLUSION

The ongoing reporting of workforce disparities faced by racially minoritised hospital staff underscores the inadequacy of current interventions in addressing negative workplace behaviours. It highlights the need for hospital initiatives to address the intersections between racialisation, racism and incivility in order to effectively progress towards an inclusive, positive work culture. Moreover, there is a lack of comprehensive understanding regarding how covert behaviours function, contributing to this complex workforce issue. Our global scoping review examined subtle, uncivil behaviours and experiences of racially minoritised staff from both internal (staff) and external (patients and visitors) sources. It revealed that rude and disrespectful behaviours manifest in diffusive ways, leading to multi‐level consequences that significantly strain workgroup relations and impact patient care processes. As health‐care workforces diversify, through domestic and international recruitment, they can bridge gaps, align more with patient’s demographics, and enhance cultural knowledge. However, this diversity introduces challenges at interpersonal and structural levels, necessitating proactive hospital programs, projects and actions to foster inclusion and address racialised interpersonal dynamics. Given the increasing diversity within hospital workforces, there is an imminent need for transparent, multi‐pronged processes to foster perceptions of fairness and trust. These measures are crucial for mitigating the negative impacts of incivility, which heightens social tensions, disrupts information sharing in hospital teams and generates vulnerabilities in the health‐care system.

## AUTHOR CONTRIBUTIONS

All listed authors meet the authorship criteria and agree with the content of the manuscript. Olivia Joseph, Ghazala Mir, Rebecca Lawton and Beth Fylan were responsible for the study concept, design and article screening. Pam Essler supported shaping the screening, analysis and dissemination materials. Olivia Joseph and Ghazala Mir were responsible for drafting the manuscript. All authors approved the final version for submission.

## CONFLICT OF INTEREST STATEMENT

No conflicts of interest have been declared.

## DISCLAIMER

The views expressed in this article are those of the author(s) and not necessarily those of the NHS, the NIHR or the Department of Health and Social Care.

## Supporting information

Supporting Information S1

Supporting Information S2

Supporting Information S3

Supporting Information S4

Supporting Information S5

## Data Availability

The data that support the findings of this study are available in the supplementary material of this article.

## ENDNOTES

^1^
We use the term ‘racially minoritised’ to acknowledge that racial and ethnic identities are socially constructed, continuously created by people in society through dynamic social processes of power and oppression. Minoritised individuals and groups are subject to social forces that reduce their value, opportunities and advantages in society (Milner & Jumbe, 2020).

^2^
The term ‘Lay Leader’ refers to six members of the public that have a substantial research role in decision‐making processes across the research cycle within the Yorkshire and Humber Patient Safety Research Collaborative. They are partners that provide critique, suggestions and have connections to community links.

^3^
Cliques are a group of individuals that have shared interests, statuses, experiences and perspectives that can be exclusionary and which can be unwelcoming to others (Powers et al., 2014).
