# Supplementary material for: Incivility experiences of racially minoritised hospital staff, consequences for them and implications for patient care: An international scoping review
Source: Sociol Health Illn. 2024 Mar 20;47(1):e13760. doi: 10.1111/1467-9566.13760 (PMC11684503; doi:10.1111/1467-9566.13760)
Supplement: Supplementary file 1 — Supporting Information S1 [file SHIL-47-0-s005.docx]

# Supplementary Material 1: Search Strategy (Academic and Grey Literature) and eligibility criteria

## Development of the eligibility criteria and search strategy

The eligibility criteria and search strategy were shared and iteratively discussed with different members of the review team (including the lay leader and research librarian). The included lay leader actively participates on the boards of charity organisations that focus on addressing healthcare inequalities and of the six lay leaders; she expressed a keen interest in contributing to our project. She expanded the search terms (particularly for healthcare workers), refined workshop plans, and supported facilitation of interactive workshops with patients, carers and members of the public. Additionally ten racially minoritised healthcare staff informed the focus of the scoping review by sharing insights based on their lived experiences, related to the relevance, importance and benefits of the review questions. The focus on the global healthcare workforce, definitions of incivility, the educational or awareness raising outcome and dissemination style of the scoping review were informed by discussions with stakeholders (further details can be found here: <https://shorturl.at/pCU29>).

## Eligibility Criteria

| Inclusion Criteria |  |
| --- | --- |
| Population | Focus on racially minoritised hospital workers definitions, and attribution of incivility experiences within a hospital setting.  Articles will only be included if racially minoritised employees are part of the participant population and articles explicitly report analysing and describing findings related to that population. |
|  | All countries can be considered, and papers will be included where the authors themselves have defined any race or ethnicity as a minority in relation to the country's local population. |
|  | Publications within an academic setting will only be included if the participant explicitly discusses their experiences of hospital placements. |
| Concept | Incivility (including related and interchangeable concepts such as lateral violence, horizontal violence, abuse, negative behaviours, bullying, and harassment) is the primary ‘exposure’ of interest in the analysis. |
| Context | Include healthcare workers that are primarily based within a hospital setting. All countries will be included. |
| Time | All databases will be searched from 1999 to 2021. |
| Language | English language only (due to lack of funding and resources for translation). |
| Study Type | Published and unpublished original research, thesis/ dissertations and grey literature. |
|  | All qualitative, quantitative and mixed-method methodological approaches utilised to explore incivility (and related concepts). |
| Data Charting/  Extraction | For each paper we extracted the following data: Author/s, Year, Title, Journal, DOI, Country of Origin, Context - Hospital Information e.g. employee numbers, Context - Hospital Type, Context - Department or ward, Participants - Final Numbers, Participants - (Ethnicity, Race, Nationality), Sex, Participants - Job Role/s, Aim/ Purpose, Research Question/s, Methodology, Study Design, Methods, Data Analysis, Study Design - Measures or questions used, Theoretical Framework, Concept, Source of incivility (or related concept), Other source of incivility (or related concept), Authors definition of incivility (including concepts related to incivility), Population of interests definition of incivility (including concepts related to incivility), Conceptualisation, Behaviours associated with incivility (or related concept), Perception, Experience, Attributions, Circumstances - situational factors, Triggers, Consequences, Key findings, Recommendations, Future research.  Independent reviewers undertook a two-step screening process, and any disagreements were discussed until the final list of included studies.  All types of reviews were included narrative, systematic, scoping etc. IF search terms were explicitly stated and aligned to the aims and objectives outlined in this scoping review to ensure retrieved articles are relevant to the review questions. The reviews must state the population, concept and context as a focus of the review in the aims and/or introduction. |
| Data analysis | Extracted data was subject to a narrative synthesis guided by Popay (2006), which involved frequency counts, content analysis and thematic analysis dependent on the type of data e.g., quantitative, qualitative and research question. |

## Academic and Grey Literature Search Strategy

Literature were obtained from multiple sources including relevant electronic databases with international literature (Ovid MEDLINE, CINAHL, Cochrane Database of Systematic Reviews, Embase, Global Health, PsycINFO, Scopus and WorldCat). Grey literature was located via popular search engines, such as Google and limited to the first five pages of results due to feasibility and resource limitations. Due to the subject of the review, it is important to intentionally identify non-western sources within the search strategy, therefore international agencies and databases such as World Health Organisation, Global Health (Ovid), GreyNet International, and other sources identified by stakeholders were also searched for published and unpublished literature (see table 1).

Table 1 Description of the literature

| Published | Grey literature and unpublished reports |
| --- | --- |
| Cumulative Index to Nursing and Allied Health Literature (CINAHL; 1982 to present)  Ovid MEDLINE (1950 to present)  EMBASE (1947 to present)  PsycINFO (1967 to present)  Cochrane Database of Systematic Reviews  Global Health (Ovid)  WorldCat  **Hand searches:**  BMJ Quality and Safety  BMC Health Services Research  Journal of Patient Safety  Bulletin of the World Health Organization (LMIC)  PROSPERO | Google Search (five pages of the results)  World Health Organisation  OpenGrey  GreyNet International  ProQuest |

Search strategies were tailored based on the electronic database. The search strategy including all search terms for the Medline Ovid database are provided in table 2.

Table 2 Example search strategy for Medline

| Search Strategy – identify search terms, synonyms, variant spellings, MeSH terms | | | |
| --- | --- | --- | --- |
| PCC Framework | Term | Free Text – Synonyms/Alternative Words | MeSH Terms/ Subject Headings |
| Population | Racial and ethnic minority  Healthcare Workers | (ethnic minorities OR Black, Asian and minority ethnic OR Black, Asian and ethnic minorit* OR minoritised OR nondominant OR minorities OR racialised OR visible minor* OR ethnic group* OR ethnic population* OR minority group* OR minority population OR racial and ethnic minorit* OR people of colour OR BIPOC OR indigenous OR Divers* OR Migrant OR racial identit* OR ethnic identit* OR “socially ascribed ethnicity” or “socially ascribed race” or “socially ascribed identity” AND  Healthcare workers or healthcare staff or healthcare professional or doctors or physician or senior house officer or sho* or registrar or consultant or medic or intern or fy1 or fy2 or resident or specialist or nurses or surgeons or midwifes or healthcare assistants or allied healthcare professionals or ((hospital) adj2 (staff or employee* or personnel or worker* or receptionist* or secretar* or manager* or porter* or assistant* or aide* or attendant* or orderly or orderlies or auxiliar*) | [**Minority Groups**](https://www.cochranelibrary.com/advanced-search/mesh#0); **Ethnic Groups**  **Health Personnel; Allied Health Personnel; Health Workforce; Medical Staff** |
| Concept | Incivility | (Incivility OR Rude* OR Discourteous OR Disrespect* OR unprofessional OR “negative behavio?r” OR “negative behaviour*” OR Aggression OR violence OR bullying OR abuse OR lateral violence OR “interpersonal conflict” OR conflict OR “bad manners” OR “interprofessional relations” OR micro-aggressions OR micro-invalidations OR micro-assaults OR micro-incidents OR micro-inequit* OR gossiping) | **Incivility; workplace violence/; Aggression/; Bullying/; antagonistic behaviour** |
| Context | Hospital | Healthcare OR healthcare setting OR, hospitals OR medical teams OR clinical teams OR acute hospitals OR hospital units OR medical centres OR general hospital*OR acute hospital* | **Hospitals/; Hospital/; Hospitals, General/** |

## Further searches

Citation tracing were used to search the references of identified articles, as well as hand searches of the relevant journals to identify materials not identified via electronic database searches due to indexing errors. Where required, authors within relevant fields for the review will be contacted directly or via research community websites e.g., ResearchGate for further information or to request access to materials not available via traditional electronic databases or web searches.
