# Supplementary material for: Incivility experiences of racially minoritised hospital staff, consequences for them and implications for patient care: An international scoping review
Source: Sociol Health Illn. 2024 Mar 20;47(1):e13760. doi: 10.1111/1467-9566.13760 (PMC11684503; doi:10.1111/1467-9566.13760)
Supplement: Supplementary file 2 — Supporting Information S2 [file SHIL-47-0-s002.docx]

# Supplementary Material 2: Collaborative analysis

## Illustrative example of collective categorising of uncivil behaviours

We aimed to understand the types of uncivil behaviours described by racially minoritised workers. We utilised a basic qualitative content analysis, as described by Popay 2006, which occurred over three stages outlined below:

1. Using the Andersson and Pearson incivility definition, 159 behaviours were extracted from the 32 included articles, which were reviewed by RL, BF, GM and refined through discussion. If the behaviours were severe, overt, and clearly intentional or violent they were removed. 152 behaviours remained and were grouped into 33 categories.
2. We delivered two collaborative sense-making workshops involving self-identified racially minoritised workers and patients, carers or relatives with experience witnessing uncivil behaviours towards hospital staff. During the workshop, the 33 uncivil behaviour categories were shared, and the group were asked to pick their top 3 most important behaviours based on their own definition of incivility (Figure 1). The feedback was utilised to inform further refinement of the uncivil behaviour categories into 10 categories. During the first workshop, collaborators (racially minoritised staff and patients, carers or relatives) were shown definitions of incivility. They were asked to review the 10 categories encompassing uncivil behaviours and reach a consensus on the categories (Figure 2).
3. They shared their expertise based on their lived experience and categories were further refined into eight categories in a subsequent session (Figure 3).

## Figure 1: Example of the workshop activities (split into two groups – staff and patients, carers or relatives)


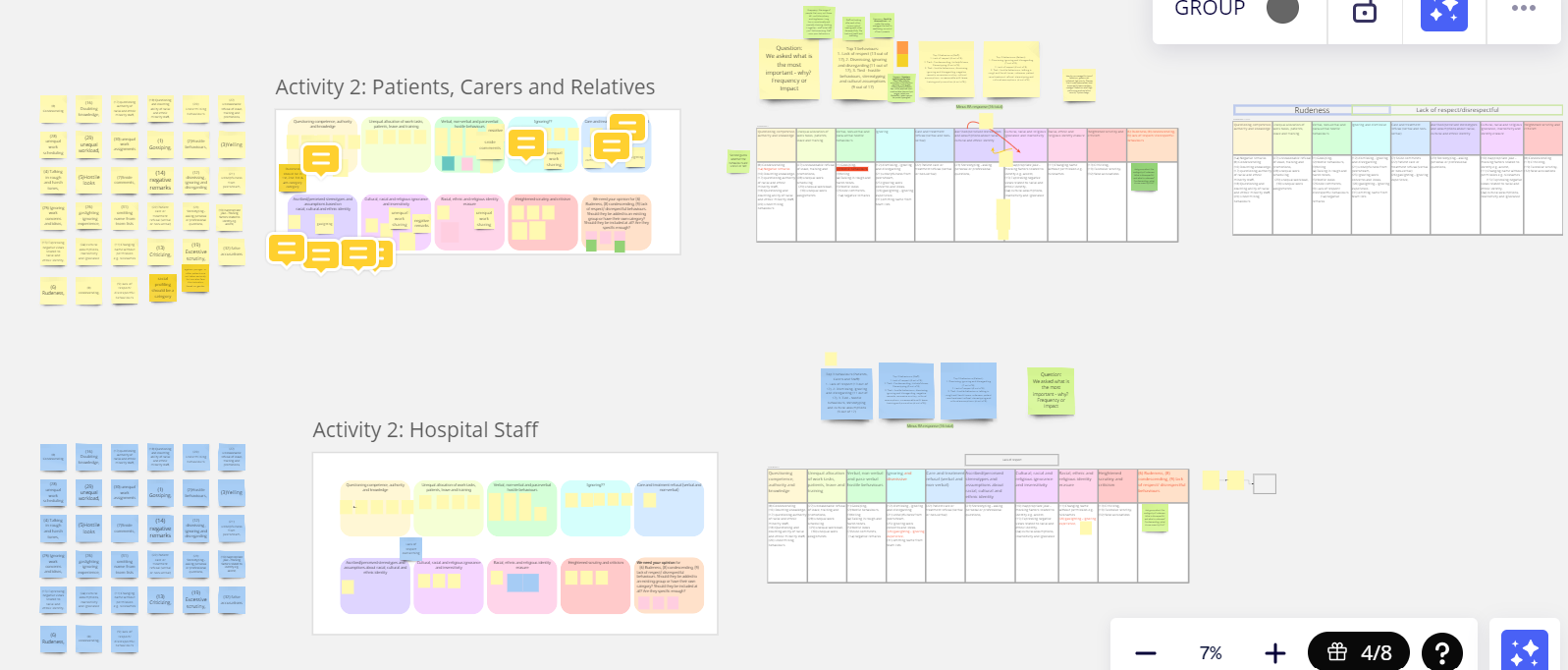

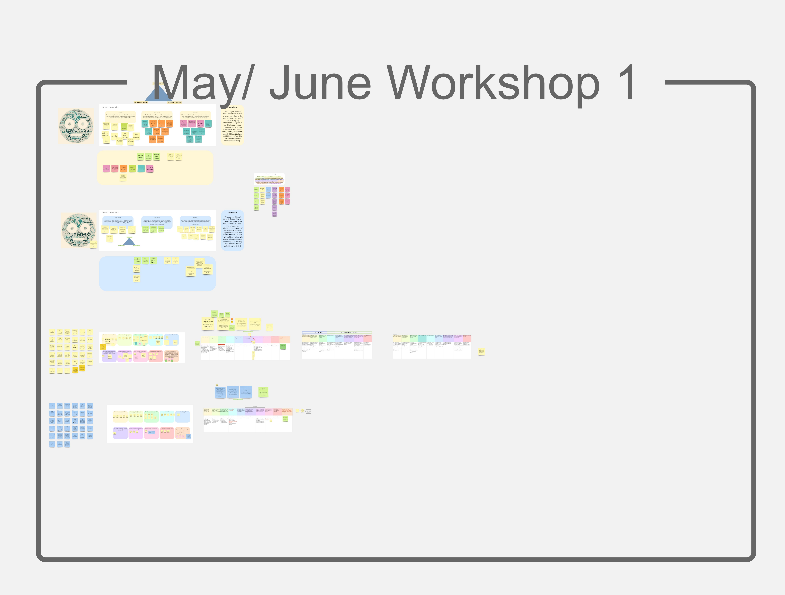

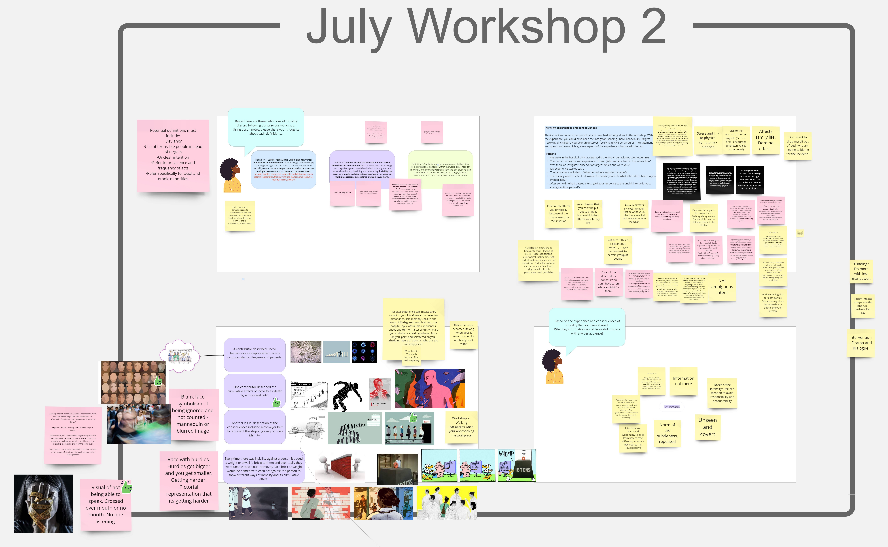


## Figure 2: Example of the online grouping activity – Ten categories


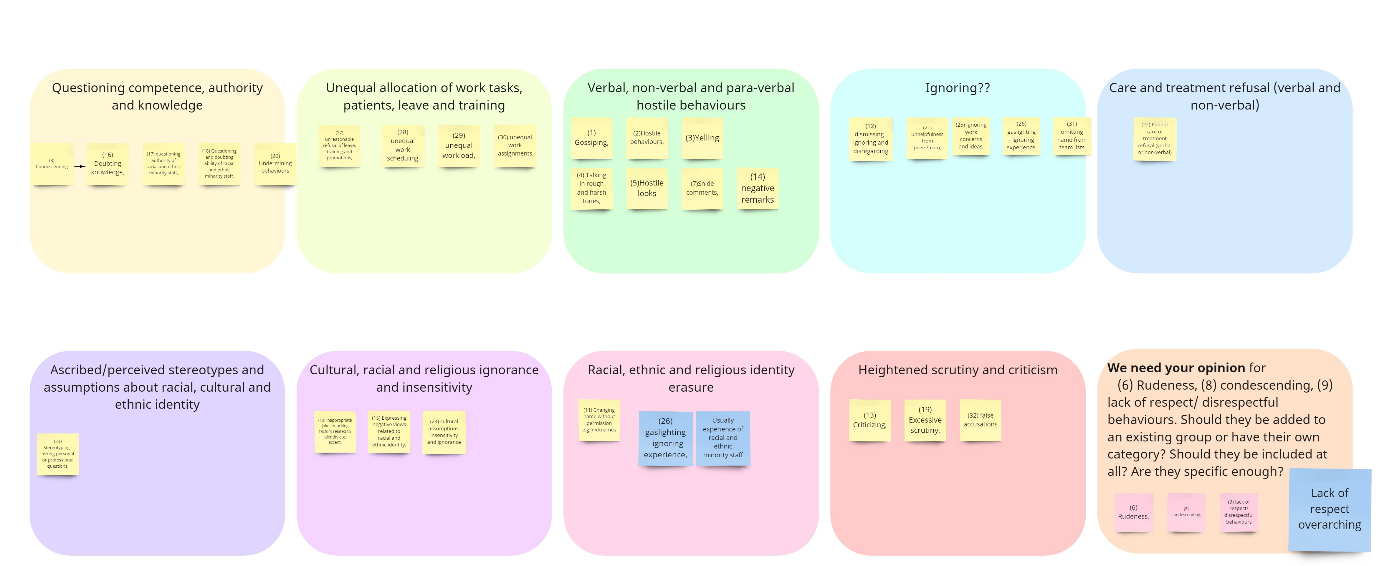

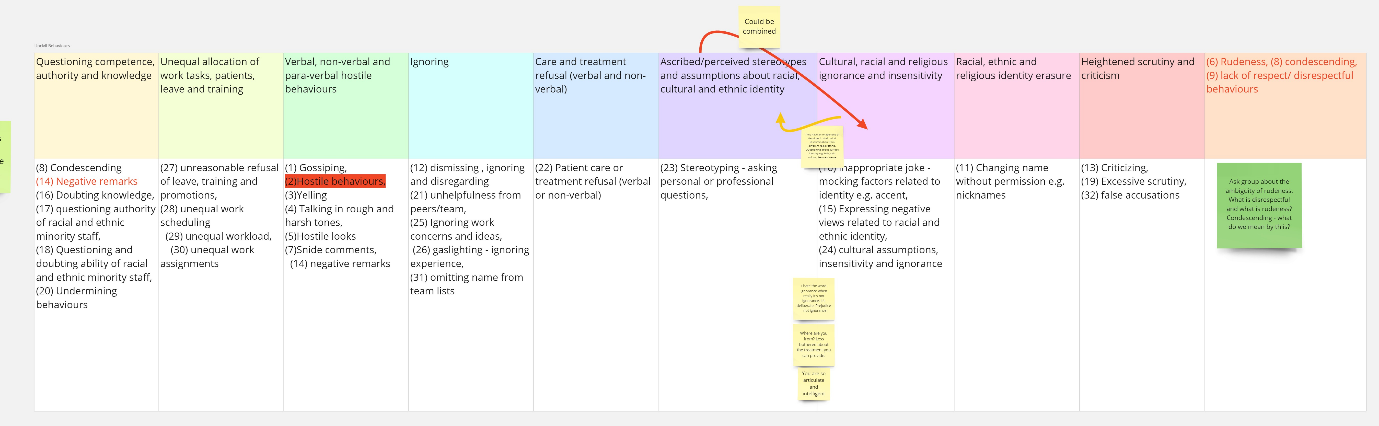


##
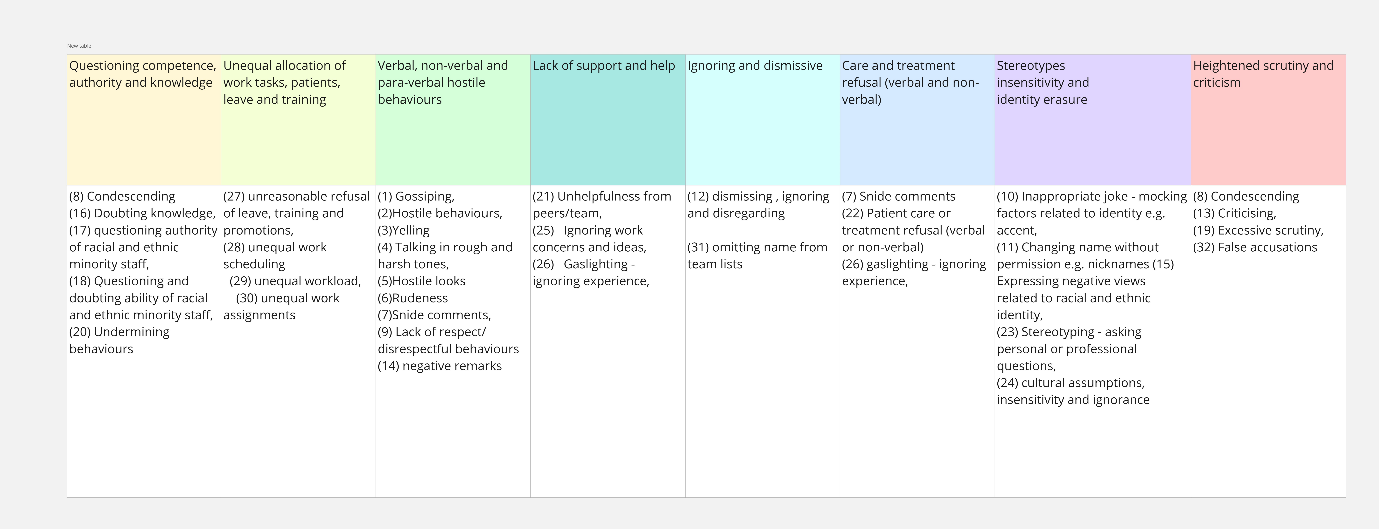
Figure 3: Final eight categories of uncivil behaviour
