# Supplementary material for: Incivility experiences of racially minoritised hospital staff, consequences for them and implications for patient care: An international scoping review
Source: Sociol Health Illn. 2024 Mar 20;47(1):e13760. doi: 10.1111/1467-9566.13760 (PMC11684503; doi:10.1111/1467-9566.13760)
Supplement: Supplementary file 3 — Supporting Information S3 [file SHIL-47-0-s001.pdf]

NIHR | National Institute for Health and Care Research

UNIVERSITY OF LEEDS

TACT eThnicity, rAce and inCivility in hospiTals

What uncivil behaviours do racially and ethnically minoritised hospital workers experience?

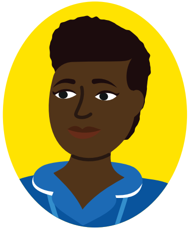

Incivilities are originally defined as "low-intensity poor behaviours with ambiguous intent to harm the target in violation of workplace norms for mutual respect".
Andersson and Pearson 1999

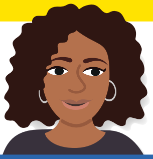

Background

We looked at current evidence* across the world to understand the uncivil behaviours that racially and ethnically minoritised hospital staff face at work. We found eight commonly described behaviours.

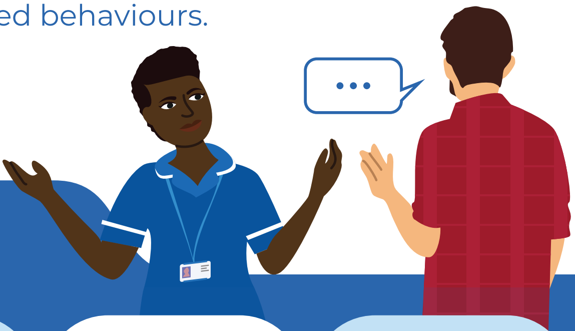

What are the eight categories of uncivil behaviours?

- **Verbal, non-verbal and para-verbal hostile behaviours**
- **Stereotypes insensitivity and identity erasure**
- **Unequal allocation of work tasks, patients, leave and training**
- **Questioned competence, authority and knowledge**
- **Indirect refusal of care or treatment**
- **Unsupportive and unhelpful behaviours**
- **Ignoring and dismissive behaviours**
- **Increased scrutiny and criticism**

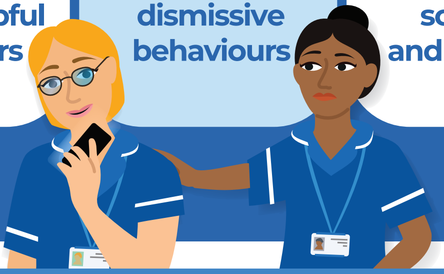

*Please note: We conducted a systematic scoping review which returned 2737 relevant articles of which 32 articles were included.

What are racially and ethnically minortised hospital workers' experiences of incivility?

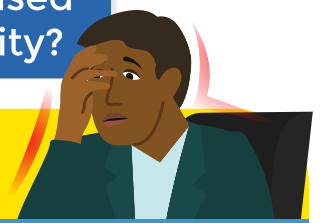

Four major themes of incivility experiences

Cultural control

Experiences of exclusion and mistreatment by their co-workers through cliques, rude behaviour and assumptions based on their culture, background or religion.

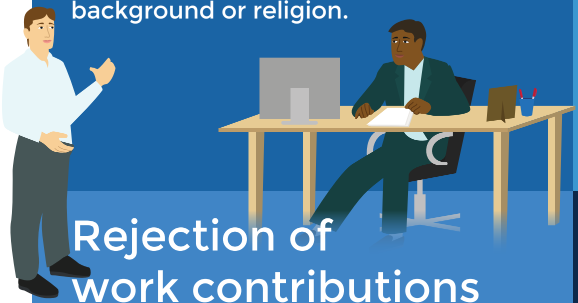

Managerial lack of concern

Nursing staff (specifically in the USA) often faced difficulties when trying to raise concerns about racial bias, uncivil behaviour from colleagues, and ideas for changes at work.

They found that their concerns were often dismissed or not taken seriously.

Rejection of work contributions

They experienced being overlooked or dismissed in their work, despite being experienced and skilled. They faced being ignored or wrongly identified.

Patients treated staff differently, for example, they were unwilling to do what the healthcare worker asked of them.

Powerlessness at work

Nursing staff and operating room technicians reported that their needs were not being met or respected by colleagues. They experienced a lack of help and support during the delivery of care.

They also shared experiences of negative talk and increased observations from colleagues.

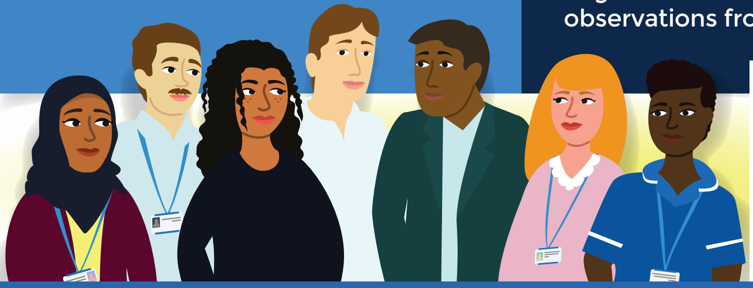

SCAN HERE

A special thank you to the TACT Advisory group
of racialised hospital workers, patients and members of the public.
For further information about the project and those involved scan the QR code.

This project is funded by the National Institute for Health and Care Research (NIHR) Yorkshire and Humber Patient Safety Translational Research Centre. The views expressed are those of the authors and not necessarily those of the NIHR or the Department of Health and Social Care.
