# Supplementary material for: Incivility experiences of racially minoritised hospital staff, consequences for them and implications for patient care: An international scoping review
Source: Sociol Health Illn. 2024 Mar 20;47(1):e13760. doi: 10.1111/1467-9566.13760 (PMC11684503; doi:10.1111/1467-9566.13760)
Supplement: Supplementary file 4 — Supporting Information S4 [file SHIL-47-0-s003.pdf]

Incivility in Hospitals

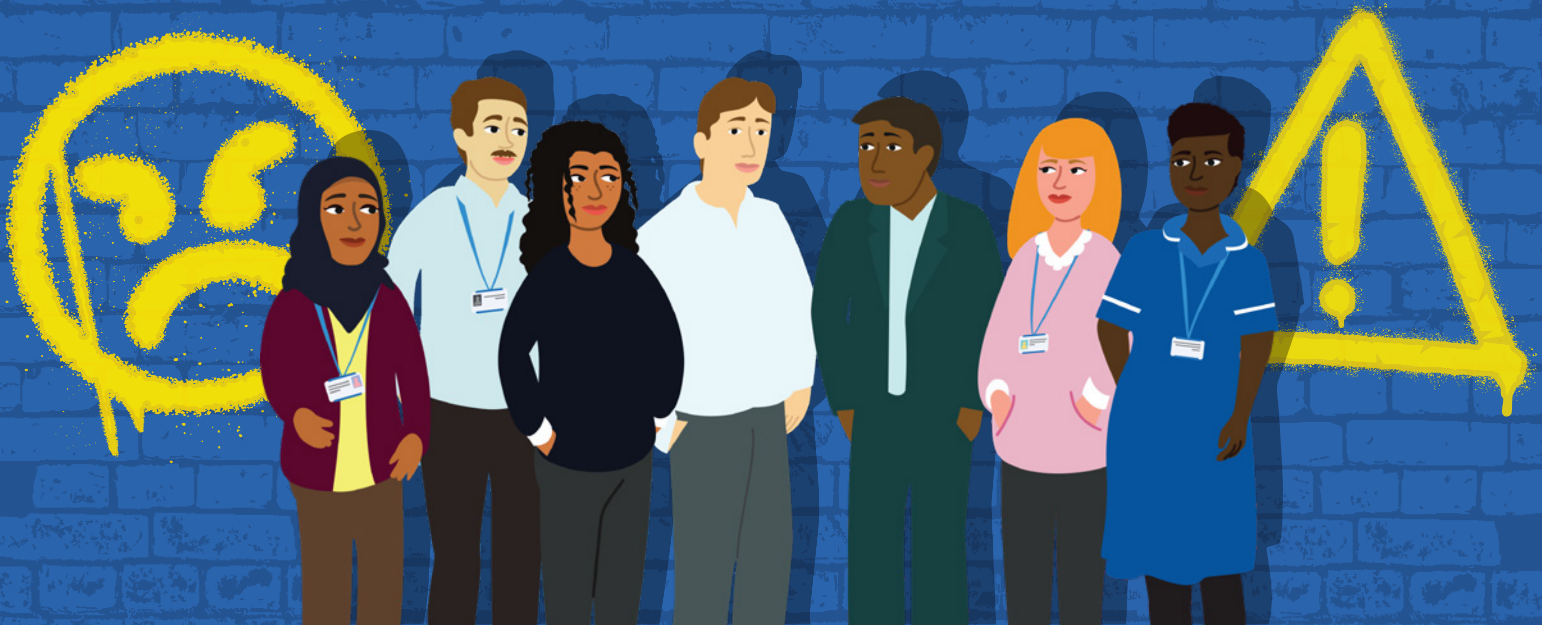

Unspoken: A Graphic Look at Incivility in Hospitals

Racially and ethnically minoritised hospital workers experience higher levels of workplace violence and mistreatment than their majority colleagues such as bullying, harassment and discrimination. However, less is known about how racially and ethnically minoritised hospital workers experience subtler, low intensity behaviours such as incivility. When people are rude or disrespectful to others at work, it can be a form of "incivility". Uncivil behaviours can seriously affect individuals, teams, and the overall functioning of healthcare organisations. It can negatively impact workers mental health and wellbeing and put patient care at risk.

The purpose of this graphic story is to show and explain the various types of incivility in the workplace, and to encourage readers to reflect on how their own words and actions can affect others. It also seeks to raise awareness of behaviours, experiences and impacts of incivility that are often unseen or ignored. It is important to be aware of these subtle behaviours and actively improve the work environment for everyone.

Our Approach

We conducted a study to learn about how racially and ethnically minoritised hospital workers feel about rudeness at work. We wanted to know:

- How these workers define and experience rudeness from their colleagues and patients
- What might cause others to be rude towards these workers
- The effects of rudeness on these workers, other staff, the hospital, and patient care.

To do this, we followed a set of steps to look at existing evidence called a scoping review. This helped us find all the relevant information that was available about this topic from around the world.

To find out what was already known about this topic, we looked at written documents including previous studies (written up as articles), reports, reviews, and posters. We used certain words to help us find the right information. These words were about the people we were studying (population), incivility e.g. rudeness (the concept), and the hospital setting (context).

We had a list of rules for deciding which documents to include in our review. This list changed as we had new discussions and made decisions about what should be included or excluded. We had three people check them to make sure we chose the right ones.

From **2737 articles**, we included **32 articles in our final review**.

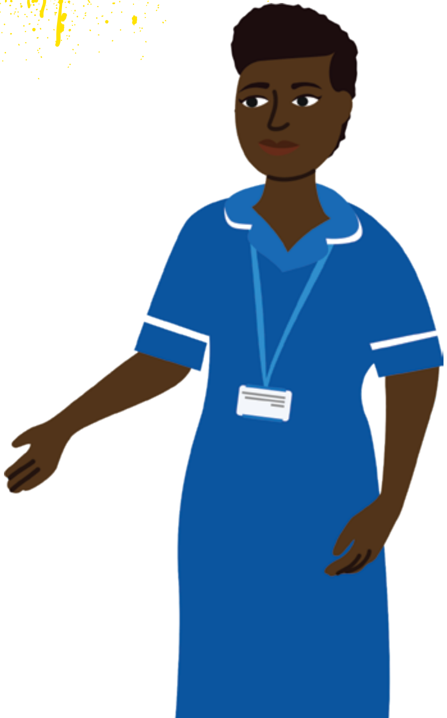

What countries were the included articles from?

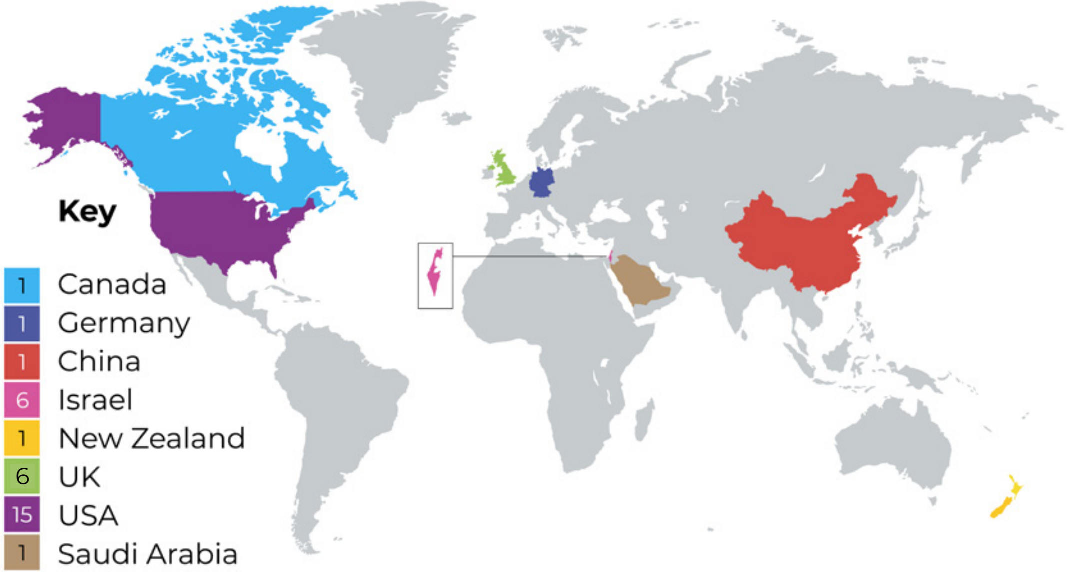

"It is too dangerous to appear as if you don't have thick enough skin or take offence at everything."

- Staff Advisory Group Member

What did we find? Results of our Review

We identified eight categories of uncivil behaviours, which are shared below. The following pages share the findings, starting with commonly identified uncivil behaviours followed by experiences, consequences and recommendations.

1. Questioning competence, authority and knowledge
2. Unequal allocation of work tasks, patients, leave and training
3. Verbal, non-verbal and paraverbal hostile behaviours
4. Ignoring and dismissing
5. Lack of support and help
6. Indirect refusal of care or treatment
7. Ascribed stereotypes, insensitivity, and identity erasure
8. Increased scrutiny and criticism

Meet the Characters

This visual short story shows a collection of uncivil behaviours and experiences, which were identified in our review of existing evidence. There are five stories with three scenes each and two key questions to consider. A short summary of the review findings will be shared in between the stories.

The story follows **six fictional characters** that cross paths across one day in a hospital setting and shows the many ways that uncivil behaviours are felt, seen, and expressed in the workplace. The story is meant to encourage readers to pause, reflect and consider the impact of their words and actions on others and to raise awareness of how incivility may affect people differently.

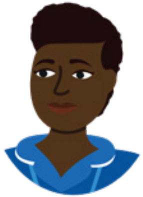

Renelle
Midwife

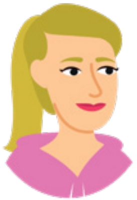

Sarah
Patient

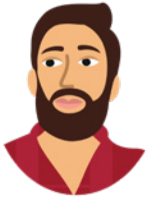

Dave
Relative

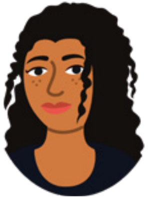

Paige
Rota Coordinator

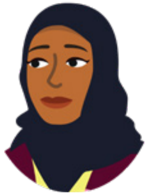

Zaynab
Receptionist

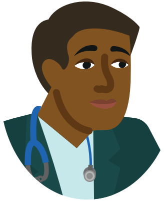

Michael
Obstetrician

Spreading Incivility

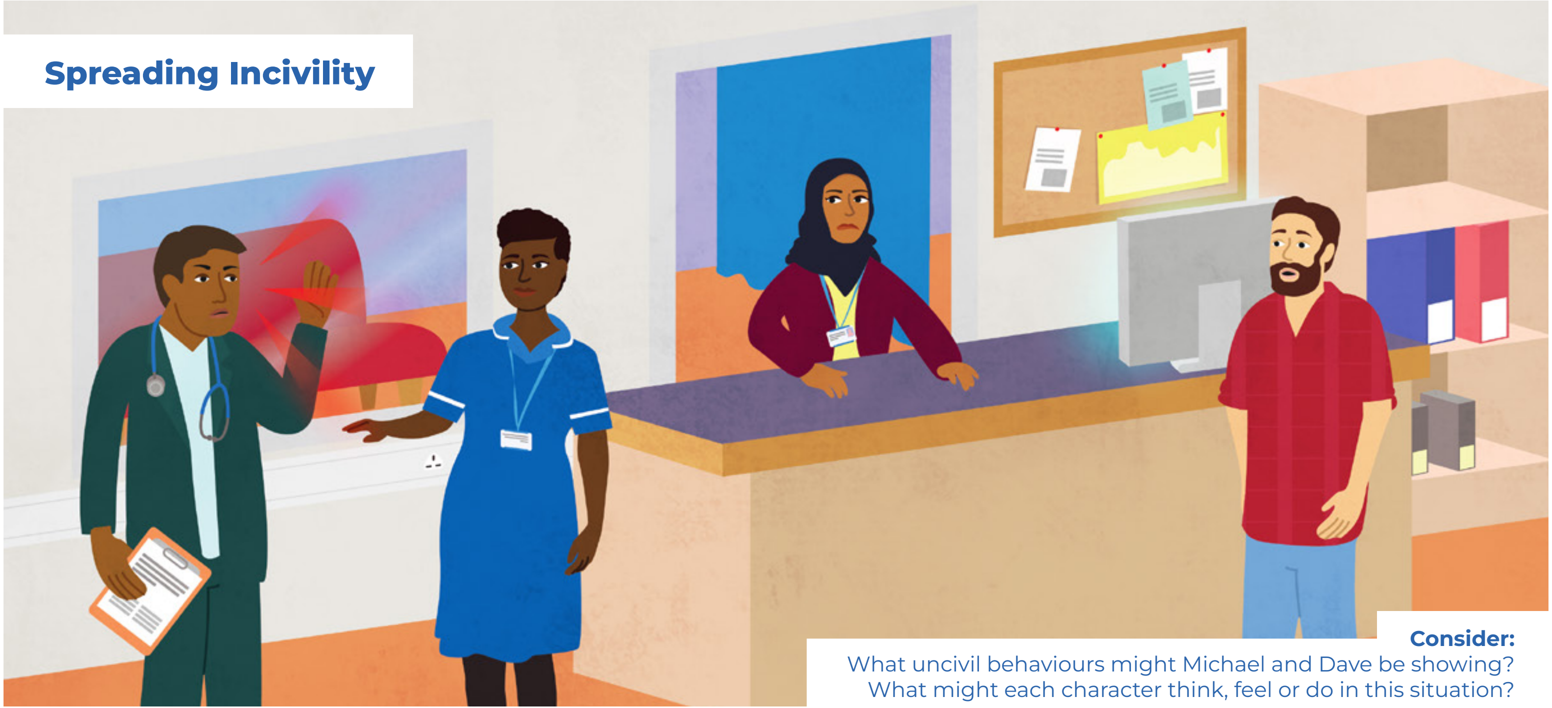

Consider:
What uncivil behaviours might Michael and Dave be showing?
What might each character think, feel or do in this situation?

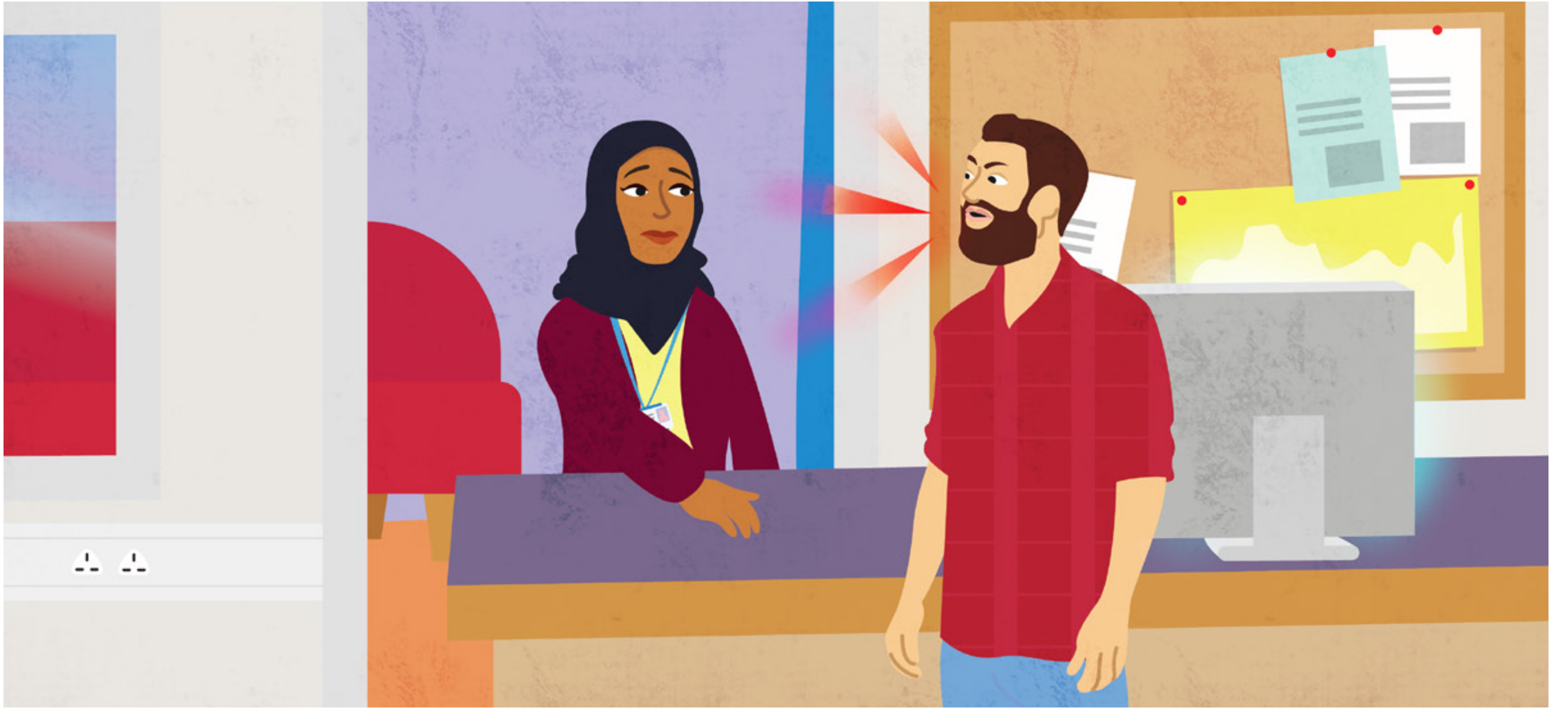

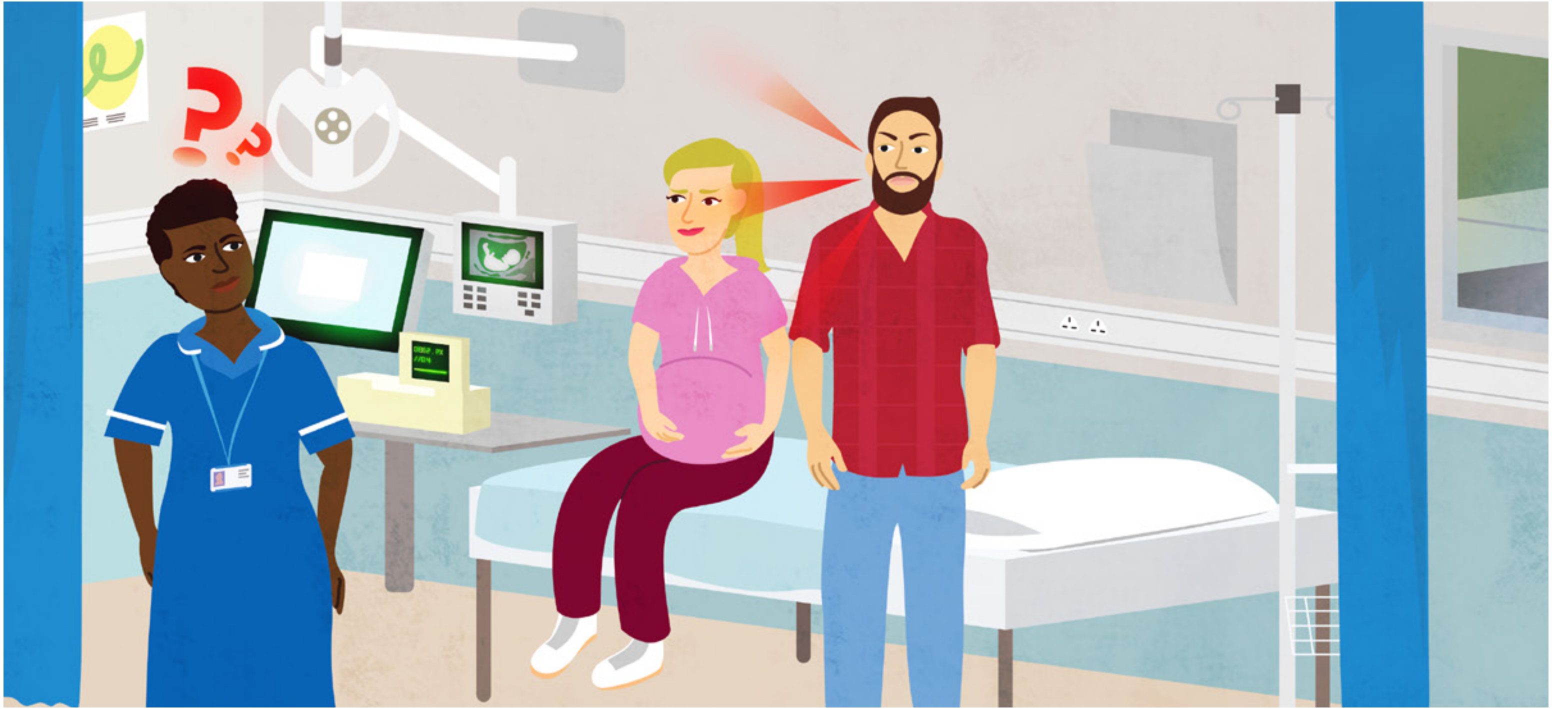

Pyramid of incivility

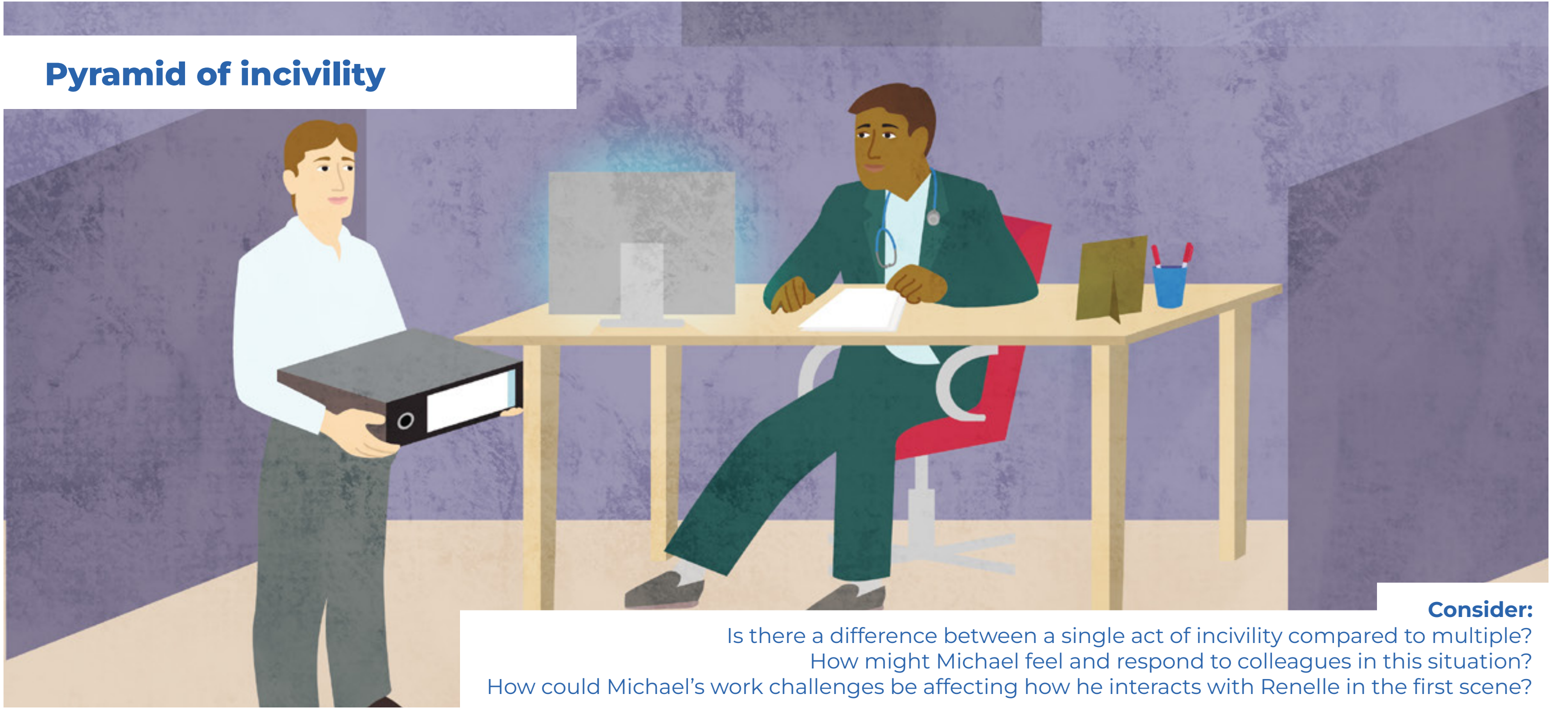

Consider:

Is there a difference between a single act of incivility compared to multiple?

How might Michael feel and respond to colleagues in this situation?

How could Michael's work challenges be affecting how he interacts with Renelle in the first scene?

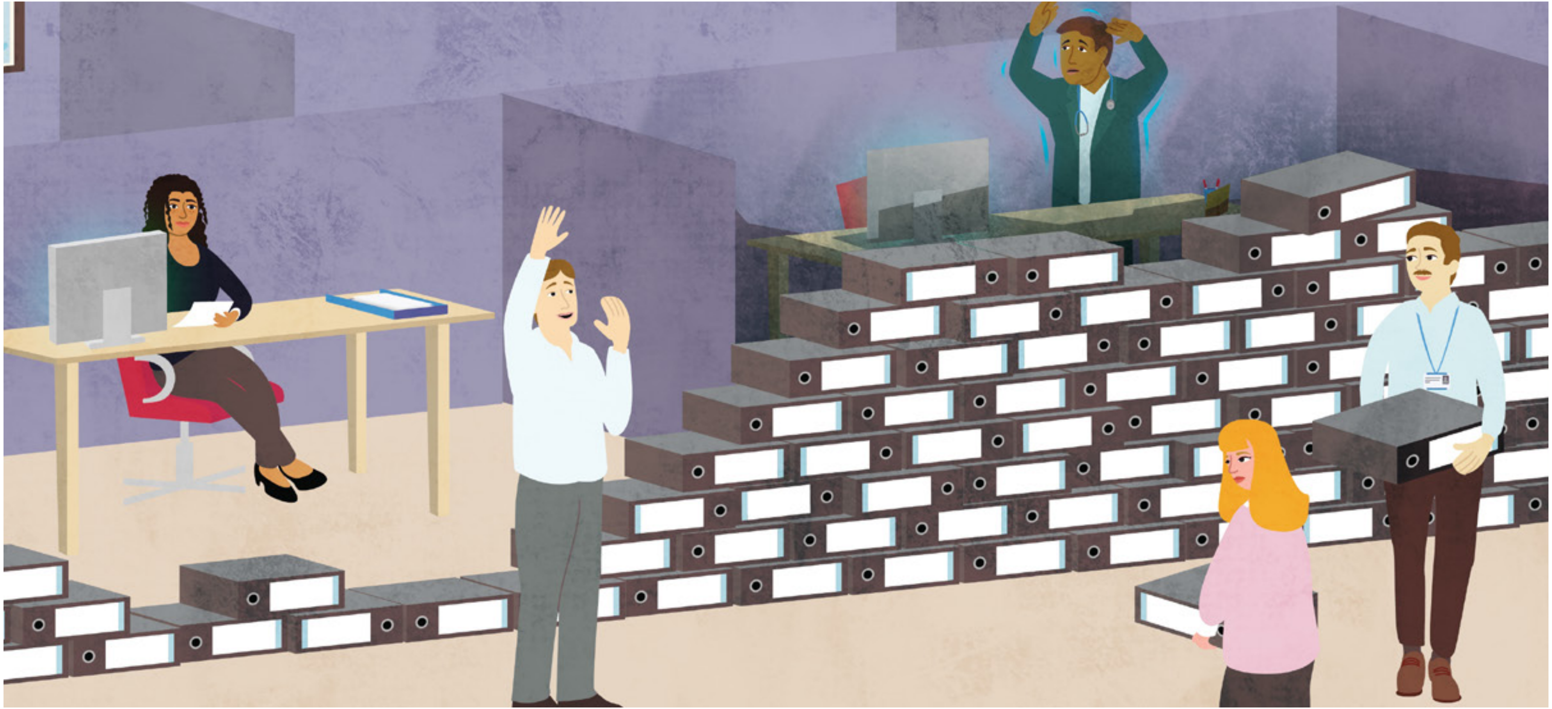

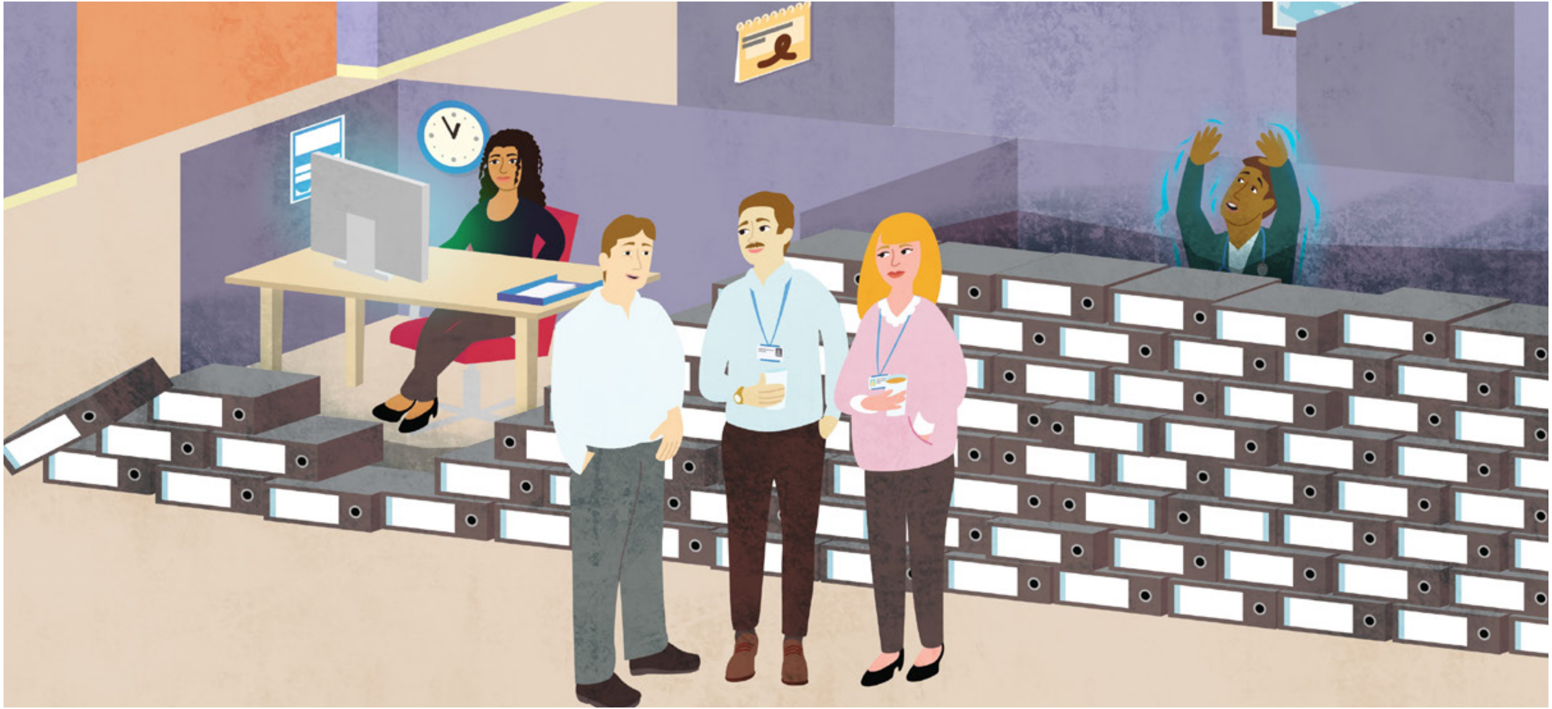

What were the experiences of incivility?

Four major themes were generated from the review: 1) cultural control, 2) rejection of work contributions, 3) powerlessness at work, and 4) manager or supervisor indifference or lack of concern about incivility.

1. Cultural control

This included experiences of exclusion and mistreatment by their co-workers through cliques, rude behaviour and assumptions based on their culture, background or religion. These behaviors made them feel unwelcome in the workplace.

2. Rejection of work contributions

They experienced being overlooked or dismissed in their work, despite being experienced and skilled. They faced being ignored or wrongly identified. Additionally, patients treated them differently, for example, they were unwillling to do what the healthcare worker asked of them.

3. Powerlessness at work

Nursing staff and operating room technicians reported that their needs were not being met or respected by colleagues. They experienced a lack of help and support during the delivery of care. They also shared experiences of negative talk and increased observations from colleagues.

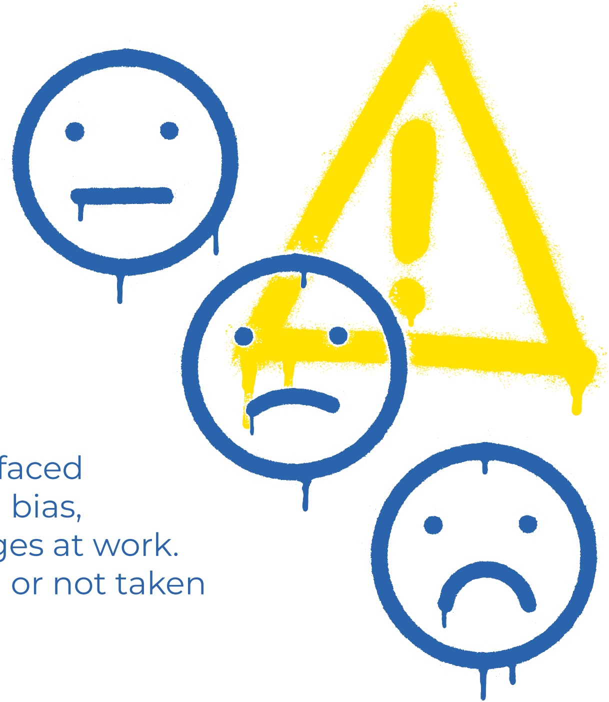

4. Manager lack of concern

Nursing staff (specifically in the United States) often faced difficulties when trying to raise concerns about racial bias, uncivil behavior from colleagues, and ideas for changes at work. They found that their concerns were often dismissed or not taken seriously.

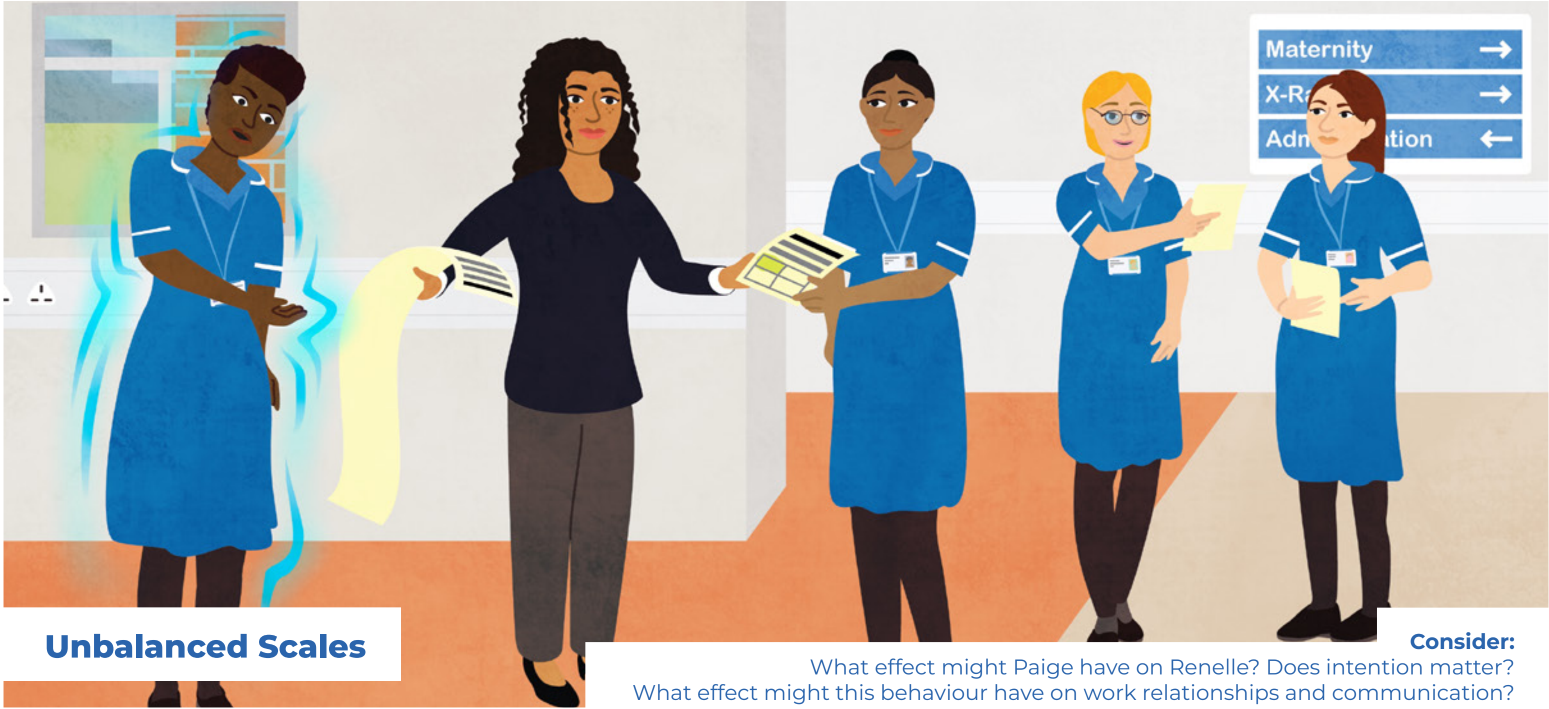

Unbalanced Scales

Consider:
What effect might Paige have on Renelle? Does intention matter?
What effect might this behaviour have on work relationships and communication?

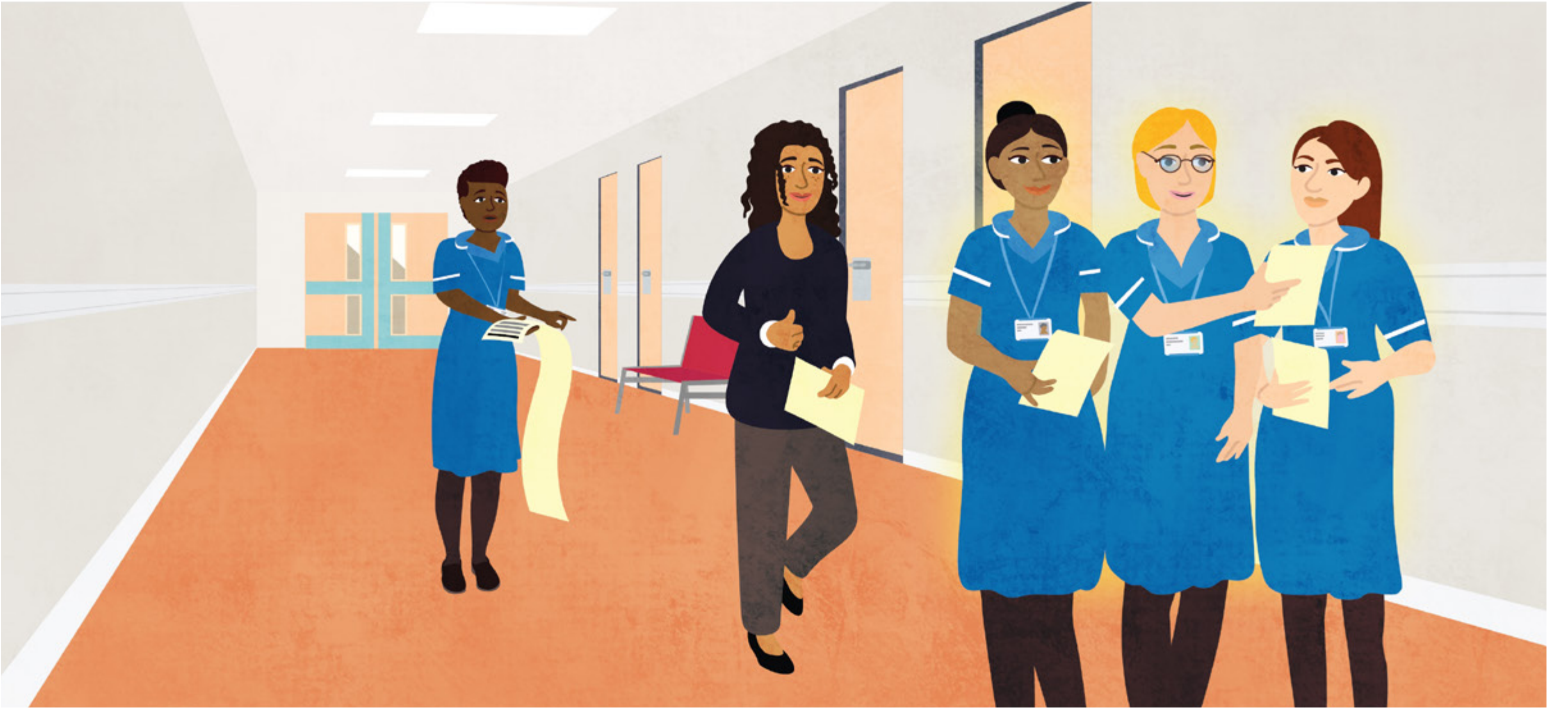

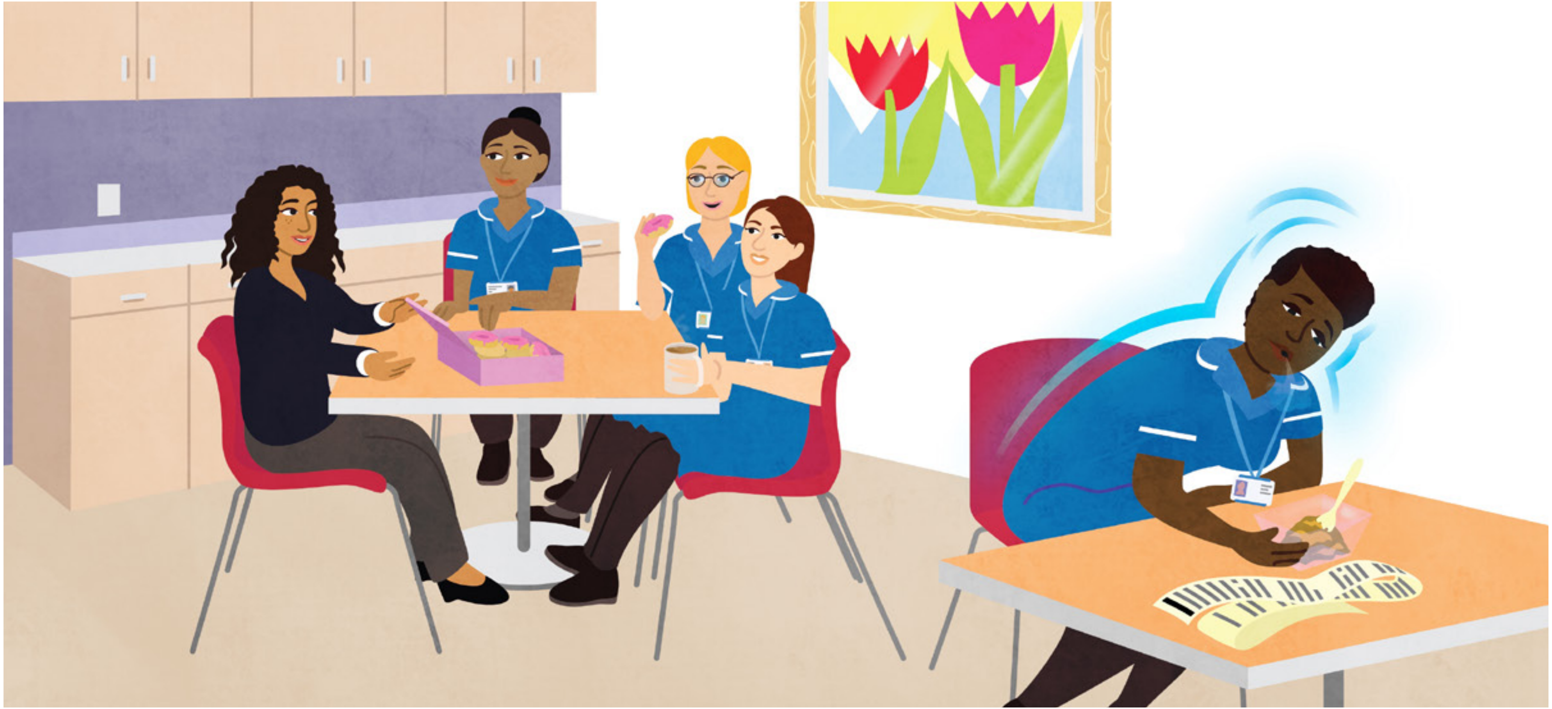

What are the consequences of incivility?

We found that when racially and ethnically minoritised hospital workers experience uncivil treatment from colleagues, it can cause negative emotions and feelings. Incivility resulted in reduced wellbeing and poorer mental and physical health.

The review found that experiences of incivility can harm relationships and communication between colleagues, reduce job satisfaction and lead to poorer care delivery.

Lastly, uncivil behaviours caused workers to think about leaving their job.

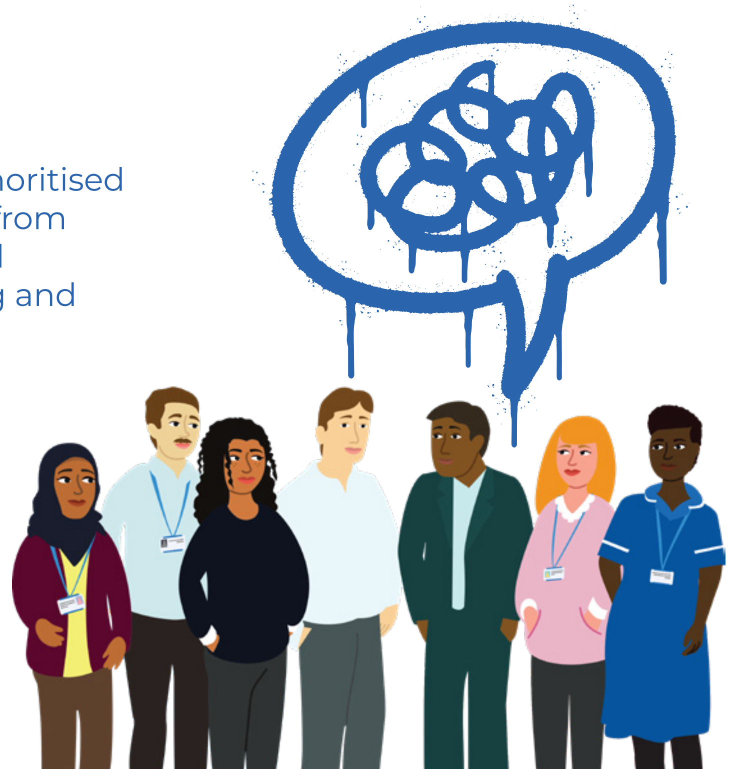

Incivility Glasses

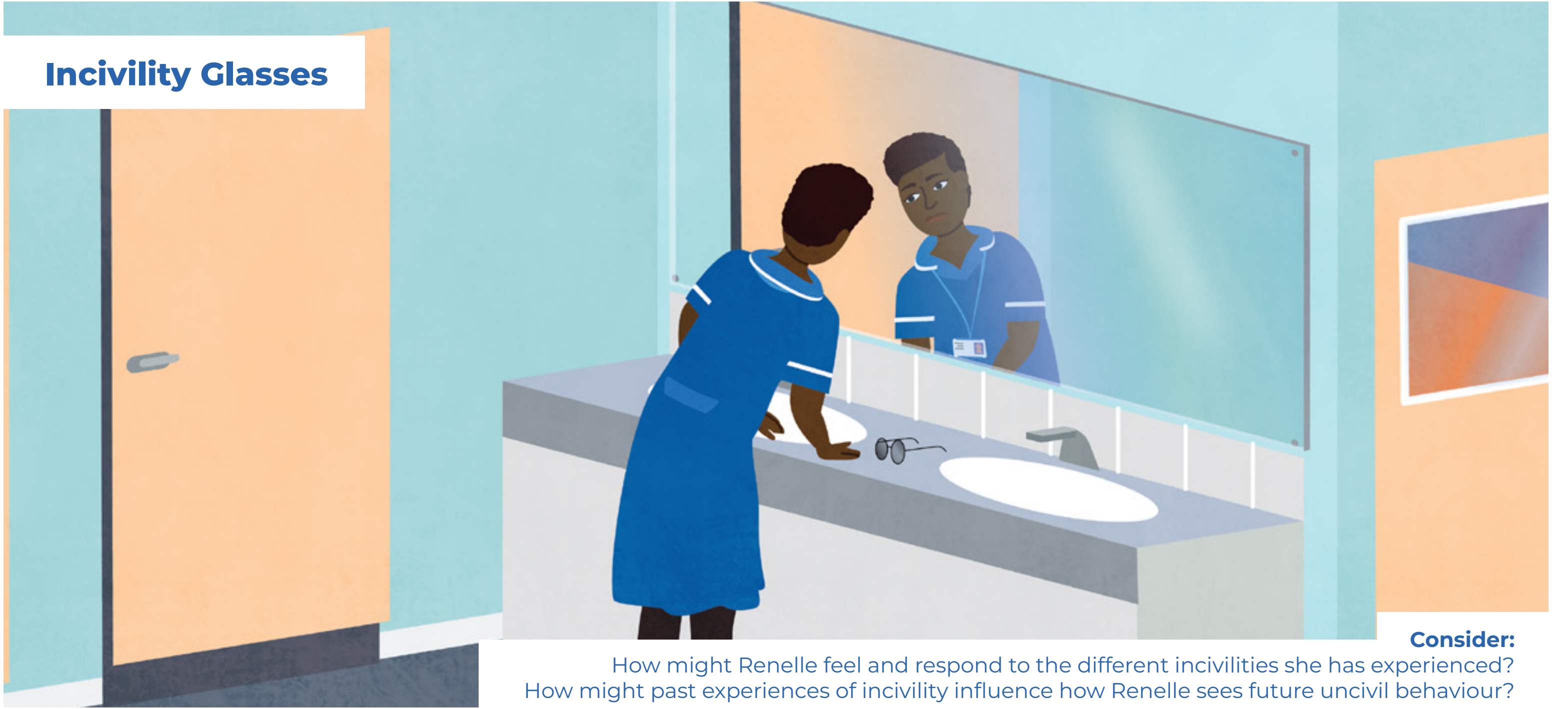

Consider:

How might Renelle feel and respond to the different incivilities she has experienced?

How might past experiences of incivility influence how Renelle sees future uncivil behaviour?

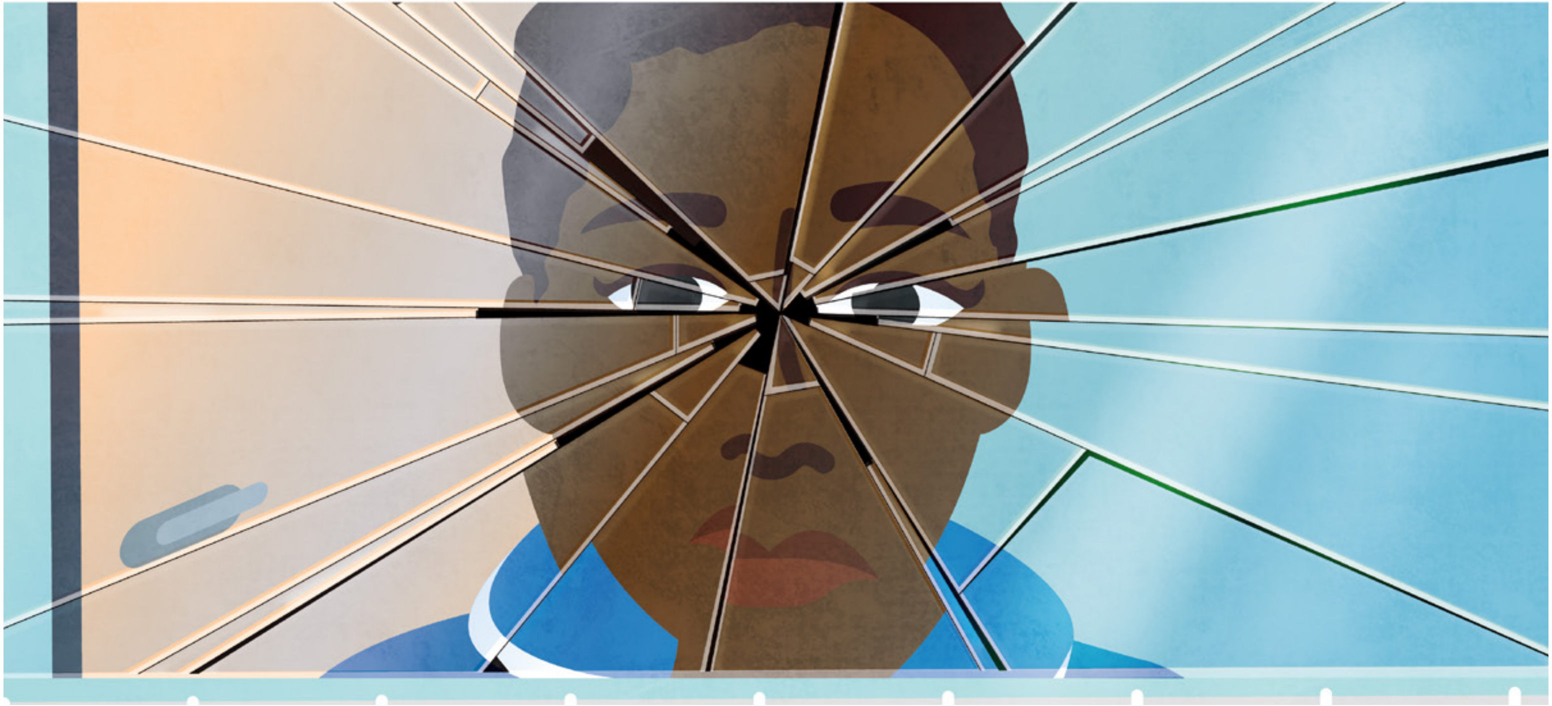

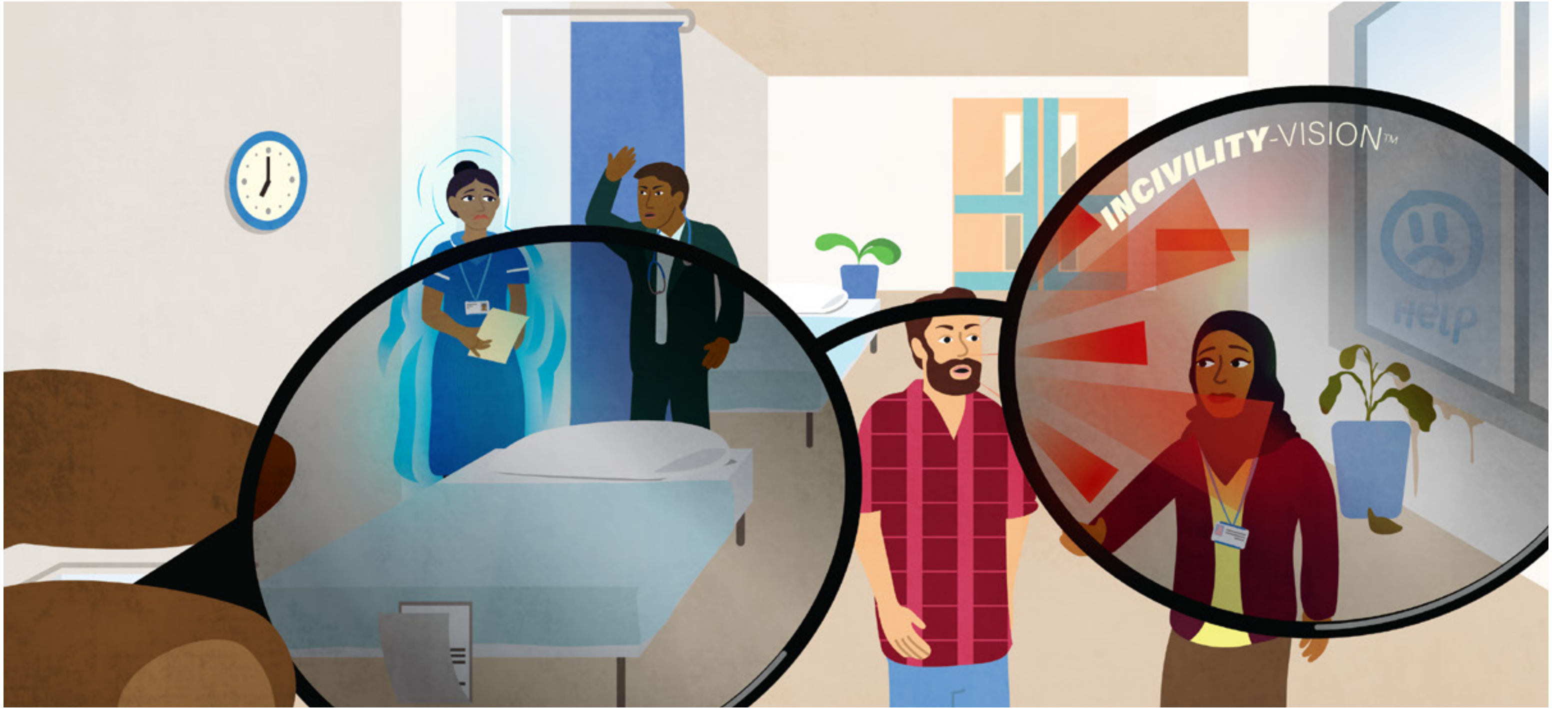
INCIVILITY-VISION™
Help

Recommendations to address Incivility

We identified five key recommendations from the authors outlining how to address subtle uncivil behaviors in hospitals. The five areas of recommendations include:

- creating inclusive environments,
- developing tried and tested solutions,
- ongoing organisational review and reflections,
- improving the work environment,
- and supporting effective leadership.

These areas highlighted the need for training for all workers to address their own biases, and required learning for leadership to recognise, acknowledge and address uncivil behaviours. It showed the importance of developing clear, fair and trustworthy reporting mechanisms, and creating shared expectations between workers at all levels, patients and visitors.

The Daily Cycle

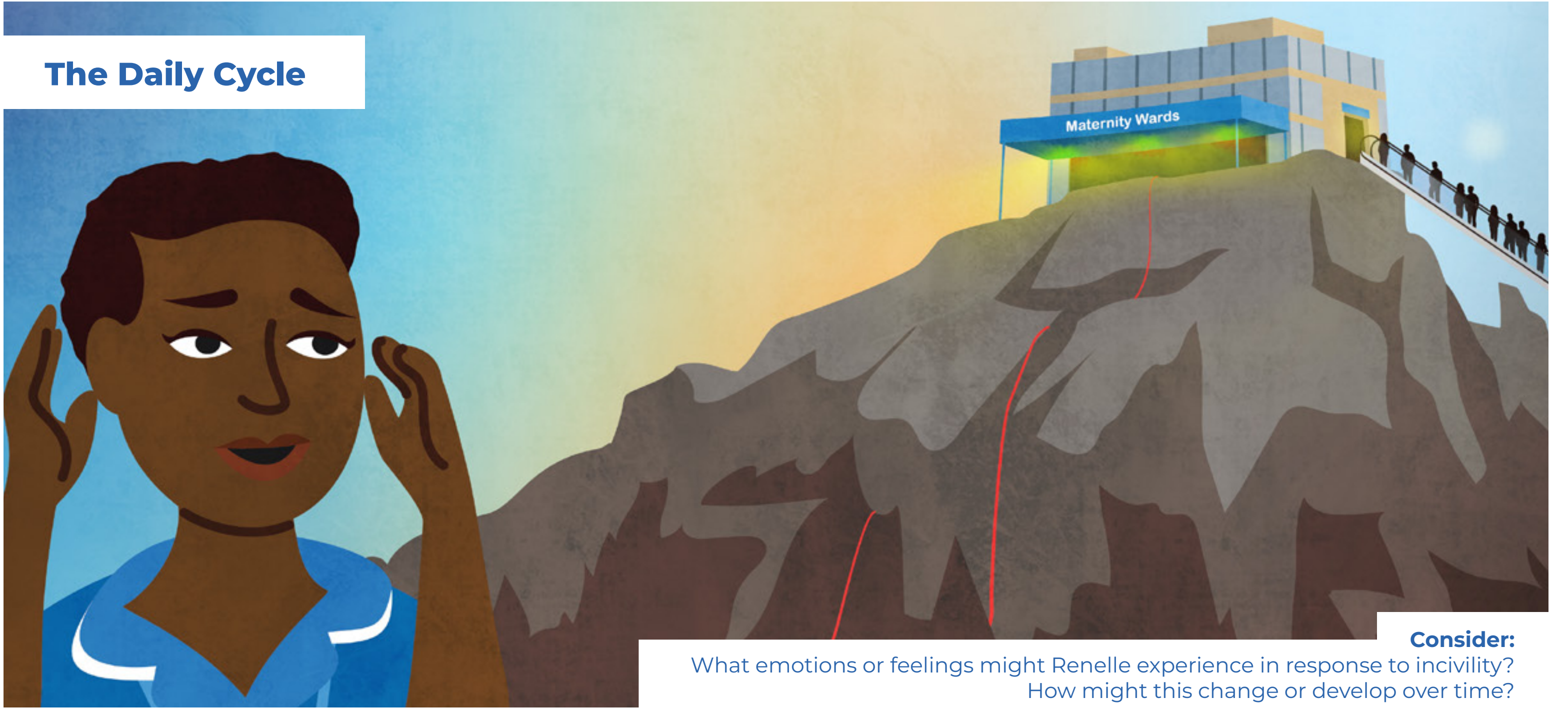

Consider:

What emotions or feelings might Renelle experience in response to incivility?
How might this change or develop over time?

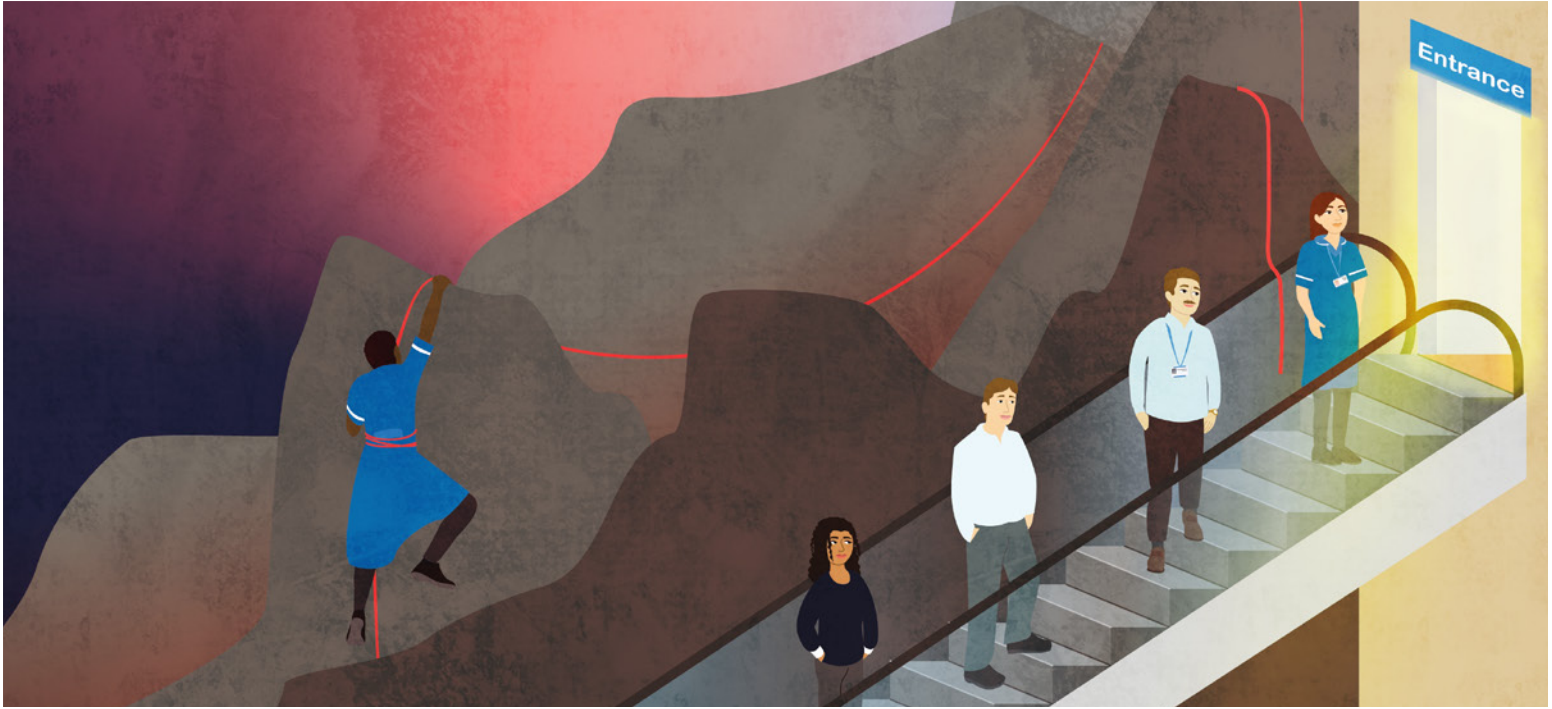
Entrance

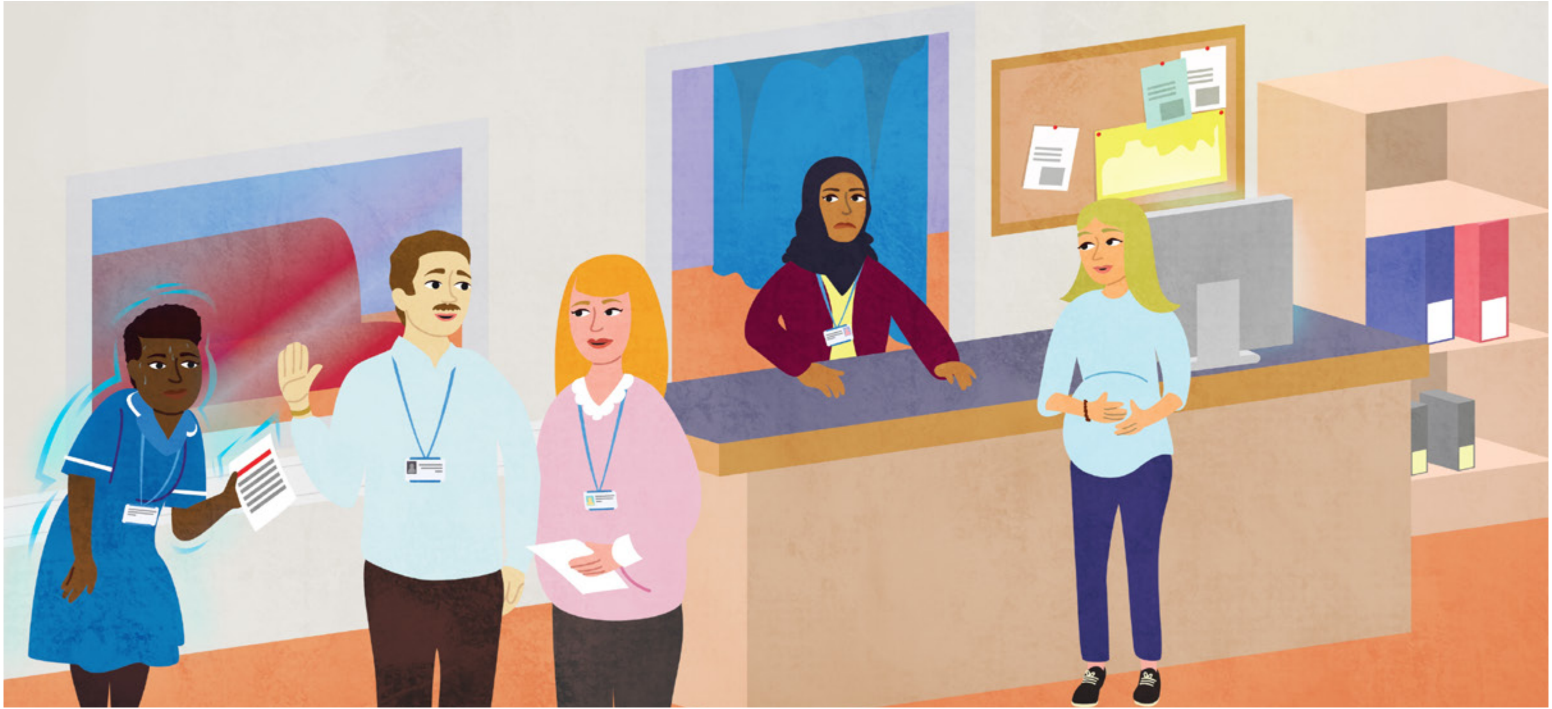

A Picture Can Paint a Thousand Words

These storyboards highlight the various ways that uncivil behaviours can occur from rude words and gestures, to being ignored and the unequal allocation of work tasks. Incivility can be experienced from lots of different sources including colleagues, managers, patients and relatives.

It can be observed by others and affect their day. It can influence the work environment and change the behaviours that are considered acceptable to be more and more negative. We hope that these stories start important discussions about the different ways incivility can show up and the impact it can have on staff, patients and the organisation.

It is important to understand how incivility affects hospital workers that are racially and ethnically minoritised to develop effective, comprehensive solutions to address them, and achieve fairness and equity for all.

Reflection Questions

Defining incivility

- How do you define incivility?
- What are the main features of incivility?
- How can it be differentiated from other types of workplace mistreatment e.g. bullying, harassment?

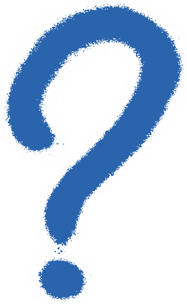

Types of uncivil behaviours and environments

- Considering the categories of uncivil behaviours, which ones are most common?
- Do they all cause the same level of harm?
- Does the frequency or the source of uncivil behaviour matter?
- Who is most likely to experience incivility?
- How might structural factors (e.g. racism, sexism) influence experiences and perceptions of incivility?

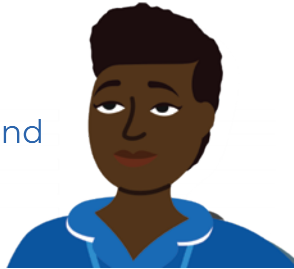

Impact of incivility

- How might incivility affect people differently?
- How do personal and professional factors such as past experiences, culture, age, gender, role, and status influence the impact of rude and disrespectful behaviours?
- How can incivility harm team relationships and performance?
- How can incivility harm caring processes and the delivery of safe care?

Solutions

- How can we raise awareness of behaviours, experiences, and impacts of incivility? Who should be involved in promoting a culture of respect and kindness?
- What can we do to encourage colleagues and managers to pause, reflect, and consider the impact of their words and actions on others?
- What tools or processes need to be in place for workers to feel safe to challenge and address uncivil behaviours such as training, fair reporting processes, mentoring?

Support for the Reader

We understand this is a sensitive topic and can cause negative emotions. The resources below have been provided for support. Please note, these materials are not a substitute for medical advice. If you have any concerns, speak with your doctor.

Resources

Confidential text service → Text FRONTLINE to 85258 for 24/7 support

Samaritans → Call 116 123 for support

Shout → Text SHOUT to 85258 for crisis text line support

Mind → Information on racism and mental health
www.mind.org.uk/information-support/tips-for-everyday-living/racism-and-mental-health

Bayo → Mental health support for Black communities: https://bayo.ubele.org
