# Supplementary material for: Incivility experiences of racially minoritised hospital staff, consequences for them and implications for patient care: An international scoping review
Source: Sociol Health Illn. 2024 Mar 20;47(1):e13760. doi: 10.1111/1467-9566.13760 (PMC11684503; doi:10.1111/1467-9566.13760)
Supplement: Supplementary file 5 — Supporting Information S5 [file SHIL-47-0-s004.docx]

# Supplementary Material 5: Characteristics of included studies (in alphabetical order)

| Article # | Author(s)  & year | Country | Aims | Participants (Number, type and  Racially and ethnically minoritised populations) | Methodology and methods | Key findings related to review questions:  Experience and Consequences |
| --- | --- | --- | --- | --- | --- | --- |
| 1 | Alshehry et al., 2018**^1^** | Saudia Arabia | To investigate the workplace incivility of nurses working in Saudi hospitals and analyse its influence of the nurses ProQol | 378 Nurses; **Minority Groups - Filipino 103 (27.2%), Indian 165 (43.7%)** | Quantitative; Cross-sectional survey | **Experiences** - Saudi nurses had more experience in nurse incivility compared with other nationalities. Indian and Filipino nurses experienced significantly less nurse incivility and patient/ relative incivility than the Saudi (majority) nurses did.  **Consequences** - Not specifically related to race or ethnicity |
| 2 | Bae et al., 2020^2^ | United States (US) | To explore the possible triggers of clinicians disruptive behaviour and to consider whether the type of trigger resulting in disruptive behaviour differed by type of clinician, clinician characteristics, professional role, and ethnic background | 1559 Nurses and Attending Physicians; 1135 reported Race/Ethnicity; **Asian 120 (10.6%), Black or African 68 (6%), Other 64 (5.6%); Race/Ethnicity Nurse total 680, Asian n=71 (10.4%) Black or African n=44 (6.5%), Other n=42 (6.2%) Race/Ethnicity: Physician total 426, Asian n=44 (10.3%) Black or African n=22 (5.2%), Other n=18 (4.2%)** | Quantitative; Cross-sectional survey | **Experiences** - Asian clinicians reported experiencing significantly less disruptive behaviours than white clinicians did.  **Consequences** - Not specifically related to race or ethnicity |
| 3 | Bagilhole & Stephens 1999^3^ | United Kingdom (US) | The present study is an examination of Equal Opportunity policies and their implementation at a single National Health Service (NHS) hospital in relation to the recruitment, career development, training and promotion of female ethnic minority employees. | 50 administrative, domestic and nursing grades; **ethnic minorities** | Qualitative;  In-depth, semi-structured interviews | **Experiences** - Unfair treatment in requests for training and sick leave, being moved to other parts of the hospital and being isolated without proper support.  **Consequences** - isolation. |
| 4 | Boateng & Adams 2016^32^ | Canada | To explore the experiences of visible minority and White nurses, including the conflicts they experience in practice. | 66 Nurses; **Visible minority (n=38, 57.6%), Immigrant (n=38, 57.6%)** | Qualitative;  In-depth, semi-structured interviews | **Experiences** - 20 out of the 38 visible minority participants reported experiencing racial abuse, excessive scrutiny, criticism and monitoring, and being excluded by their colleagues. 15 out of 38 of the visible minority participants indicated they felt they were constantly watched and monitored. Minority nurses undermined or were less likely to be listened to by white registered practice nurses and personal support workers. Colleagues that are angry (when conflict occurs) won't talk to the nurse or be helpful when delivering patient care and can affect the work.  **Consequences** - Experienced considerable stress, fatigue, sickness which led to turnover and sickness absenteeism. Negative emotions. Hesitant to ask for support. Job performance was negatively affected, and there was a lack of cohesion and co-operation amongst work teams. Patient care reduced. |
| 5 | Brooks 2016^4^ | United States (US) | The purpose of this study was to gather an understanding of the participants’ lives before, during, and after nursing school, by examining how their childhood and education, nursing education program, and professional experiences influenced their journeys to become registered nurses. | 14 Nurses; **African American** | Qualitative; Open ended to semi structured interviews | **Experiences** - African American nurses experienced behaviours including having to prove their competency, sarcasm and negative remarks when asking for assistance and social isolation from team members.  **Consequences** – The nurses described exposure to these behaviours as tiring, feeling undervalued. Rose described leaving her role with 12 others. The safety implications of conducting long medical procedures (craniotomy) without being supported to take a break. |
| 6 | Brunton & Cook 2018^5^ | New Zealand | The aim of this study was to examine the viewpoints and experience of both New Zealand qualified nurses and internationally qualified nurses in managing communication within teams and the clinical practice context in an increasingly diverse healthcare workplace in New Zealand. | 53 Nurses; **36 internationally qualified nurses (first registered in the United Kingdom (8), China (5), the Philippines (11), India (9) and South Africa (3)) as well as New Zealand Māori (2) New Zealand Pacific (2)** | Qualitative; Semi structured interviews and focus groups | **Experiences** - Misunderstandings around informal language and jokes, increased isolation and international educated nurses were suspicious that the jokes were about them. Assumptions about language skill based on foreign status. Checking and double-checking work tasks caused tensions with New Zealand colleagues, which was associated with different values related to caring responsibilities. Questioning capabilities. Offensive statements from patients and colleagues about 'common sense' and being interrupted in discussions.    **Consequences** – feelings of shame, feeling stupid, offended, loneliness, reduced confidence in competence, suspicion between colleagues, workload allocation based on patient care refusals and sacrifice preferred communication patterns. |
| 7 | Cohen 2013^6^ | United States (US) | To explore the experience of female Orthodox Jewish registered nurses. | 17 Nurses; **Orthodox or Modern Orthodox Jewish (religious minority)** | Qualitative;  In-depth, semi-structured interviews | **Experiences** - Staff asking inappropriate or insensitive cultural and religious comments, patients asking inappropriate personal comments, unfair scheduling, hostile remarks, name calling insensitive comments, being scoffed at and assumptions that Jewish workers should work all the American legal holidays, treatment of Jewish patients discussed with Jewish healthcare staff. Rude remarks and perception of unfair rules from instigators, feeling excluded, inappropriate comments to Jewish staff or witnessing inappropriate comments towards other minorities and inappropriate comments from other minority workers.    **Consequences** - Individual consequences included psychosomatic and physical symptoms e.g. anxiety, sobbing with frustration, lack of sleep, insomnia, palpitations. Feeling targeted, excluded and isolating work experiences. Organisational consequences such as leaving the job. Patient consequences – as a result of mean co-workers the Jewish staff member was short with a patient. |
| 8 | Cottingham et al., 2018^7^ | United States (US) | To explore the effects that nurses social location of race and gender have on how emotions emerge from and help to shape events within white institutional spaces and the reverberating impact they have on the nurse and their patients. | 48 Nurses; **Black (8), and Asian American (2)**. | Qualitative; Open ended audio diary | **Experiences** - Within patient care nurses reported de-legitimation of authority for nurses of colour, negative comments from patients, family members and co-workers through assumptions regarding inferiority, incompetence and selective non-compliance with treatment. Conflict with colleagues regarding scheduling, lack of teamwork, co-worker doubting and scrutinising work (Black women nurses and white nurses).  **Consequences** - Individual consequences: pissed off, upset, mad, frustrated, self-doubt, anger, and like she can “never be too comfortable.” Acknowledged additional labour of non-compliance by patients as emotionally draining, rumination, stressful situation, disproportionate amounts of emotional labour. Exhaustion and a compassion deficit. Patient consequences - depleted emotional resources negatively influence patient care. |
| 9 | Darr 2017^8^ | Israel | To explore workplace manifestations of Israel's ethnic- national struggle and to examine how Palestinian-Arab and Jewish Israeli citizens deal with the broader conflict in their daily work interactions. | 44 Nurses, Midwives, Nursing Assistants and Physicians; Hospital nurses [9 midwives]: **Arab (10)** Jewish (3) **Russian (3)**; Physicians: **Arab (3)** For example, the ‘Arab’ group included Muslim, Christian and Druze members. Among the ‘Jews’ were three nursing assistants who, though Israeli born, were of Ethiopian origin. | Qualitative; Semi-structured ethnographic interviews | **Experiences** - Accusatory looks, derogatory remarks about Palestinian Arabs and confrontation, harsh words from a Jewish Nurse to a Palestinian-Arab male nurse when reminding her that they are not enemies, unpleasant reactions from Jewish patients and families towards Arab nurses, refusal of care, and feeling less than Jewish students, annoyed Arab patient refusal to speak to a Russian nurse in Hebrew.  **Consequences** - Silence, shocked, feeling less than Jewish nurses, repression of Palestinian-Arab identity, discomfort, denigration or continued criticism of Palestinian Arab nurses. |
| 10 | Deery et al., 2011^9^ | United Kingdom (UK) | To examine the effects of harassment on job burnout and turnover intentions among hospital nurses. | 2221 Nurses; **444 minority ethnic background (20%)** | Quantitative;  Cross-sectional survey | **Experiences** - The incidence of verbal harassment was higher among minority ethnic nurses. Reported incidence of verbal harassment from managers and colleagues was significantly higher among minority ethnic nurses. Although the experience of verbal harassment was more widespread for nurses from a minority, ethnic background the effects were no different for white and ethnic minority nurses. Effective workplace harassment policies played a particularly significant role in reducing the turnover intentions of minority ethnic nurses.  **Consequences** - There was evidence to support the view that effective anti-harassment policies are important for employee wellbeing. The perceived effectiveness of the policies had a greater effect on reducing intentions to leave for nurses from ethnic minorities than for white nurses (ß = -.480). |
| 11 | Dreachslin et al., 2000^10^ | United States (US) | To explore team members' perceptions of communication processes in racially and ethnically diverse nursing care teams. | Not specified; Registered nurses, patient care technicians and support associates; **Black ethnic group** | Qualitative;  14 focus groups - separate focus groups were held for black and white employees in each of the three NCT team roles. | **Experiences** - Assumptions regarding work ethic of Black PCTs, Black PCTs treated differently to White PCTs by White RNs. People sit with their own race during lunch and discuss work leading to social isolation at work. Tense communication across difference and Black support associates expressed feeling isolated and invisible to RNs. SAs often bonded with one another, but not with the RN or PCT, SAs do not feel like important members of the team and their names are left off the patient boards by RNs and PCTs. Treatment or care refusal from Jewish patients towards Black RNs, patients use of condescending language and assumptions by patients of unskilled labour. Treated differently based on role. Unfair patient assignment, some Black SAs observed Black PCTS are allocated more patients than White PCTs. Inappropriate personal questions based on subtle stereotypes and unfounded assumptions. Differential treatment, condescending patients, unfair assignment allocation and racial assumptions can all lead to tension and conflict for black employees.  **Consequences** - Poor team work, miscommunication, isolation between colleagues. Strong association between racial diversity and difficulties with communication and conflict resolution in teams. |
| 12 | Floyd 2020^11^ | United States (US) | The purpose of this study is to explore how nurses who represent minorities share their lived experiences of incivility, bullying, and empowerment in the workplace. | 15 Nurses; **21% American Indian/ Alaskan Native, 11% Asian/Pacific Islander, 54% Black or African American, 6% Hispanic and 8% Multiple Ethnicities** | Qualitative;  The Workplace Incivility Survey was used to identify minority nurses who have experienced incivility. Followed by in-depth semi-structured interviews. | **Experiences** - Pointing out hair in front of co-workers treated as 'other', hearing racist remarks from co-workers about patients from the same racial group including culturally insensitive, assumptions or stereotypical comments. Raising a complaint or issue resulting in silent treatment, exclusion, lack of acknowledgement, checking up on work, gossiping about colleagues behind their backs and colleagues assumptions and stereotypes about minority populations, favouritism and unfair work allocations, negative response to a nurse educating physicians about care standards, and implicit biases.    **Consequences** - Avoidance of specific shifts but does not mention hospitals. Perceived to negatively affect work performance, stressful, insulting, humiliating, scarring experiences, resignation - most minority nurses leave within the year. Feeling bullied and wanting to leave. |
| 13 | Iheduru-Anderson et al., 2021^28^ | United States (US) | To report the results of a study investigating the impact of race on the work environment for African Born Black Nurses (ABBN) in the US, and how these experiences contribute to their evaluations of the healthiness of their work environments. | 17 Nurses; **Nationality: Nigeria n= 9, Ghana n=2, Kenya n=3, Cameroon n=2, Uganda n=1** | Qualitative;  In-depth group and individual interviews | **Experiences** - Not learning the nurses’ names. Lack of adequate communication, given incomplete information that requires further questioning and being ignored by colleagues. Ignored, shut down, tuned out, dismissed and interrupted by colleagues, doctors and nursing assistants due to African accent. Doctor assuming nurses do not have the intellect to communicate patients need. Spoken to rudely. Lack of performance feedback from managers and important information communicated via gossiping. Unit managers are treat African nurses with disrespect, not listening or giving time to African nurses but chatting and laughing with white nurses and aides. Managers preferential treatment of White nurses and white aides to African nurses. Colleagues teaming up against African nurses leading to social isolation from the team and lack of help from management. Complaint of perceived racial bias or discrimination not taken seriously.    **Consequences** -Negatively affected the nurses' ability to learn and perform well on the job. Undermined their self-confidence and inhibited cooperation with colleagues. In some instances, it led to withdrawal, which essentially stopped or derailed communications. Feeling scrutinised and hypervigilent. Feeling like outsiders and no belonging to the team. Mistrust and silencing of views and concerns, reduced help-seeking behaviour. Leaving the job. Burnout and job dissatisfaction. Some experienced physical symptoms related to work such as back pain, headaches, feeling of dread. Toughing it out. Reduced mental health leading to seeking therapy. Suppressed feelings and denial. |
| 14 | Jia et al., 2020^12^ | China | To examine workplace violence (WPV) towards healthcare professionals in a multi-ethnic area in China, including prevalence, influencing factors, healthcare professionals response to WPV, expected anti-violence training measures and content, and evaluation of WPV interventions. | 2036 Physicians n=624 (30.6%) Nurse n=869 (42.7%) Medical Technology n=294 (14.4%) Others n=249 (12.3%); **Minority n= 325 (16%)** | Quantitative;  Cross-sectional survey | Experiences - Ethnic minority healthcare professionals may be more likely to suffer from psychological violence, less likely to order the perpetrator to stop or report to superiors than Han people. Women of ethnic minority were more likely to suffer from psychological violence.  **Consequences** - Not reported. |
| 15 | Johnson et al., 2019^13^ | United Kingdom (UK) | To investigate the relationships between workplace bullying, perceived discrimination, levels of burnout and patient safety perceptions in nurses and midwives and to assess whether bullying and discrimination were more frequently experienced by Black, Asian and minority ethnic than White nurses and midwives. | 528 Nurses and Midwives; **Asian (15.4%), African-caribbean (2.2%), Mixed ethnicity (1.4%), Other ethnicity (0.4%), Preferred not to say (0.4%), Missing (0.7%)** | Quantitative;  Cross-sectional survey | **Experiences** - A higher rate of BAME participants (17.6%) reported experiencing bullying in the previous year compared with White participants (12.4%). The odds of experiencing bullying were 1.5 times higher for BAME participants (non-significant). Experiences of discrimination were three times more common in BAME than White nurses and midwives; 20.5% of BAME participants reported experiencing discrimination at work in the previous year compared with White participants 7.8%. The odds of experiencing discrimination were three times higher for BAME participants (significant).  **Consequences** - Bullying and discrimination have an indirect relationship with patient safety perceptions that is mediated by burnout. Both bullying and discrimination were significantly associated with higher burnout. Higher burnout was in turn associated with poorer perceptions of patient safety at both the individual and ward level. |
| 16 | Keshet & Popper-Giveon 2016^14^ | Israel | To examine the work experiences of ethnic minority Arab nurses in Israeli public hospitals. | 13 Nurses; **Arab** | Qualitative;  Semi structured in-depth interviews | **Experiences** - Stereotypical and offensive attitudes on the part of patients and their families, and tense working relationships with Jewish colleagues. Treated with suspicion, hostile looks or glances and patronised by patients. The stereotypical views, hostile glances and refusal of treatment that the Arab nurses describe are impacted by the pervasive violence that characterises the Jewish-Palestinian conflict in the Middle East. Sometimes tensions arise among the mixed teams, in which Arabs and Jews work together under a stressful workload. Tense, uneasy interpersonal interactions between colleagues leading to bad relations on the ward. The wider political situation increases fears, defensive behaviour and feeling uncomfortable.    **Consequences** - Bad relations on the wards between Jews and Arabs. Uneasy interpersonal interactions. Tense working relationships. Staff leave, withdraw into shell, feeling unsafe, feeling uncomfortable, helplessness, undermine their confidence and cloud their mood. Anticipatory fear of patient reaction or acceptance of providing care. |
| 17 | Keshet & Popper-Giveon 2017^15^ | Israel | To study manifestations of racism and discrimination experienced by Arab physicians and nurses during their ongoing work in Israeli hospitals. We examine these experiences and their interpretations and attempt to delineate the various levels of everyday racism: the micro-level of face-to-face racism; the meso-level of institutional discrimination and the macro-level of discriminatory policy and legislation. | 23 Physicians and Nurses; **Arab** | Qualitative;  Open ended In-depth interviews | **Experiences** - Hostility and rejection of Arab nurses working on paediatric wards instigated by relatives of the patient. Being yelled at, insulting remarks about Arab patients to Arab nurses, threatened by family members that want their relative not be put with an Arab patient. Suspicion, fear and hostility from patients on the wards. Different attitudes towards Arab Physicians, that are held to a higher standard than white counterparts e.g. some departments and wards check where you come from (suspicion and questioning) and sensitive relations between colleagues especially when seeking progression or moving to a new ward. Those who write the policies may hold implicit biases that are translated into policies and effect Arab Physicians at the micro-level, interactions with those that are enacting the policies.    **Consequences** - Humiliation, fear and anger, concerns going to work, uncomfortable, isolating, controlling or suppressing personal feelings or emotions, feeling responsible for reducing confrontation and minimising it. |
| 18 | Keshet et al., 2017^16^ | Israel | To assess tensions and coping strategies among ethnic minority and majority healthcare workers, to point to the differences between ethnic minority and majority healthcare workers, and to shed light on the unique experiences and perceptions of minority healthcare professionals. | 59 Nurses (n=39, 66%), Secretaries (n=5, 8%), Sanitation (n=4, 7%), Physicians (n=3, 5%), Other (n=8, 14%); **Arab (n=14 - 24%)** | Mixed Method (1);  Survey design with qualitative and quantitative questions | **Experiences** - Arab practitioners felt patients were uncomfortable, were cursed by a patient or family member and felt like a member of staff treated them badly because of their ethnic affiliation.    **Consequences** - Uneasy at work. Although Arabs are more aware of management’s support against racism than are Jews, they are more likely to feel subjected to harassment by colleagues and discrimination by management with regard to promotion and honouring their rights, owing to their ethnic affiliation. |
| 19 | Larsen 2007^17^ | United Kingdom (UK) | To examine empirically and in-depth how discriminatory attitudes and practices are experienced by overseas nurses and how the discrimination may affect their well-being and career progression and, furthermore, to apply the theoretical perspective of embodiment in understanding these processes | 2 Nurses; **Zimbabwe and Nigeria** | Qualitative;  In-depth semi-structured interviews | **Experiences** - Patients, visitors and colleagues have bypassed the Black staff nurse to talk to White student nurse or prefer to talk to the White student Nurse. Treated as invisible. Personal and professional competencies are questioned and undermined.    **Consequences** - Reduced confidence, become withdrawn and silenced, lack of trust. Feeling drained and reduced motivation to progress despite competence and skills. Emotionally disturbing experiences that affected Victoria's personality, sense of self and motivation for career progression. |
| 20 | Mitchell 2020^18^ | United States (US) | The purpose of this qualitative phenomenological study was to explore the perceptions of African American registered nurses as they transition to the nursing workforce. | 10 Nurses; **African Americans** | Qualitative;  Semi-structured interviews, open-ended questions | **Experiences** – Disrespected by other nurses (co-workers) which is observed and copied by other staff (Tech) and attributed to being new, young and treated like they are beneath them. Higher expectations for African American nurses compared to white nurses reflected in different levels of understanding issues. Ignored and not supported when having a stressful, high workload shift, despite the white supervisor being aware and other nurses were able to have a break whilst the Nurse missed her lunch. White nurses were allocated 'better' patients than Black Nurses, being given a hard time and lack of teamwork. Teamwork as an issue.    **Consequences** – Stressful- lack of respect as a stressor, inadequate or poor teamwork or helpful behaviour, breakdown in team relationships or relations between colleagues, feeling of drowning and difficulty keeping composure ‘almost exploded throughout the night’, left the hospital. |
| 21 | Moceri 2012^19^ | United States (US) | The purpose of this study was to measure the prevalence of bias in the nursing workplace as experienced by Latino(a) nurses. | 111 Nurses; **Latino(a)** Fifty six percent spoke Spanish as their first language and 31.2% reported speaking English with a Spanish language accent | Mixed Method (2); Correlational survey design | **Experiences** - 73.6% (n = 81) experienced bias and 26.1% (n = 23) perceived bias at least three times a week, 60.2% at least monthly and witnessing bias 81.8% (n = 90) in the workplace with 37.4% [n = 34) at least three times per week and 69.2% (n = 63). Latin(o) nurses reported hearing negative comments about one's race or ethnicity with 25% at least three times per week or more and 53.8% at least monthly. Patient refused care from staff due to race at least once 33% (n=36). Perceived moderate or lower levels of support.    **Consequences** - Both experiencing and witnessing bias is significantly correlated with experiencing lower levels of support and those who experience low support to staying a shorter time in the work setting. Significant link between hearing negative comments about ones racial/ethnic group and intention to stay in the workplace. The more experiences of bias Latino(a) nurses perceived, and the more they perceived low levels of support, the more likely they were to have plans to leave the workplace sooner than those who either experience low frequency bias or who perceive higher levels of support. |
| 22 | Moceri 2013^25^ | United States (US) | The purpose of this descriptive study was to answer the research question, How is bias experienced in the nurse practice environment by Hispanic nurses? Additional aims were to determine the prevalence of bias and to create an operational definition of bias in the nurse practice environment. | 111 Nurses; **Hispanic** | Mixed Method (2); Open ended survey | **Experiences** - High percentages of Hispanic nurses experienced and witnessed bias in the workplace - 73.6% (n = 81) of nurses reported experiencing bias, and 81.8% (n = 90) of participants reported that they had witnessed bias. The idea of respect, or a lack of it, became an overarching concept located within the findings. Mistaken for other roles (e.g. housekeeping /aides) by colleagues and patients as well as not being acknowledged by administrators on the unit contributed to feeling invisible. Hispanic nurses were negatively visible to supervisors when issues occurred and treatment was considered harsher than their majority (White) colleagues. Excluded from patient care and culturally relevant suggestions for Hispanic patients were ignored. Differential treatment with workload allocation. Patients and families made biased comments about the nurses’ ethnicity, skin colour, or accent, and even occasionally refused care. Colleagues made comments about Hispanic patients as if the Hispanic nurse was not present or was not Hispanic. Increased questioning and scrutiny from patients and staff, having to continuously prove themselves to fellow nurses, doctors and patients and having their competency underestimated. Those that spoke English with an accent felt more scrutinised than those without. Treated as a group rather than an individual.    **Consequences** - Tiring, stressful and painful - Described as ‘ Very painful’ ‘I get tired’ 'can be very stressful for Hispanic nurses'. Feelings of invisibility and negatively visible. |
| 23 | Onyeibe 2021^30^ | United States (US) | The purpose of this study was to explore the experiences of ethnic African nurses working in public hospitals located in the South Western United States. | 10 Nurses; **Native-ethnic African immigrants, Nigeria, Kenya, Sierra-Leone, Ghana, Egypt, and South Africa** | Qualitative; Semi-structured open-ended interview | **Experiences -** Incivility and lack of cultural sensitivity marked the overall experience of all the participants. Hyper scrutinised and unfair treatment compared to other races when they make a mistake. Subtle expressions of refusing care from nurses of colour or nurses with accents. Supervisors that are condescending and have a short attention span for black people when struggling to explain. Rude remarks and facial expressions from patients attributed to accent and colour. Communication with colleagues, patients and customers is fraught with displeasure because they made the participant felt no sense is made while talking. Interaction with hostile people. Supervisor showed a lack of respect telling the nurse that she walked around like she knew better than others. Bossy supervisors giving direct requests and not listening to ideas proposed by staff. Dismissive of authority and use of work group cliques to undermine the African American nurses authority. Unfair work allocations and undermining authority in disciplinary process. Cultural differences in communication style – perceived to be yelling at people.    **Consequences** - Intention to quit, frustration, embarrassment, and impaired communication between colleagues. |
| 24 | Pierce 2018^31^ | United States (US) | To explore African American nurses’ professional experiences within the context of the healthcare environment. | 8 Nurses; **African American** | Qualitative; Open ended In-depth interviews (face to face and telephone) | **Experiences** - Excess scrutiny from colleagues, being yelled at, not listened to and lack of respect for female nurses from physicians, rude remarks and communication from doctors to females nurses when making suggestions for patient care. They shared that their voices were not being heard within the interdisciplinary team. Common experiences of being ignored by physicians and medical students. The participants experienced being ignored and not being respected. Also, harsh words, being ignored and berated by clinicians or not listening to the needs of the patient communicated by the nurse caring for them. Dismissed concerns regarding new procedures. Testing new employees by unfair work assignments.    **Consequences** - Lack of confidence in own abilities, frustration, compromising patient care by not listening to the nurses – e.g. asking for a standing weight despite the nurse specifying that the patient could not stand or delayed access to medication by ignoring the requesting nurse, leave the organisation. |
| 25 | Popper-Giveon & Keshet 2020^20^ | Israel | To examine the experiences of healthcare workers who are members of a minority ethnic group in the context of a violent conflict. | 50 Nurses, Managers and Physicians; **Arab** | Qualitative; Semi-structured indepth interviews. | **Experiences** - Wider conflict widens differences of opinion between Arabs and Jews and spoke Arabic between colleagues evokes strong emotions from Jewish colleagues screaming that they must not speak their language in the hospital. Arab practitioners expressing opinions in support of the Palestinian struggle causes arguments between colleagues. Arab physicians feel like their opinions are silenced, and feeling pressurised to fit into the expectations of the dominant group - be the 'good Arab', 'nice Arab'. Dissociating from their personal life to be promoted.    **Consequences** -Feeling silenced. To deal with the reactions of their peers, they often choose to keep silent, hide their opinions, suppress emotions and avoid confrontation. Evokes harsh emotions. Poor team relations due to harsh emotions. |
| 26 | Popper-Giveon & Keshet 2018^21^ | Israel | To explore how individual health care professionals employed in Israeli public hospitals experience and cope with patients who refuse treatment by certain practitioners because of their ethnic origin. | 50 Nurses, Managers and Physicians; **Arab** | Qualitative; Semi-structured indepth interviews. | **Experiences** -Many mentioned having encountered incidents of treatment refusal based on a practitioner’s ethnic identity with experiences and witnessed incidents affecting colleagues. Incidents of treatment refusal are encountered more by nursing staff, students, and interns than by respected physicians and senior managers. Often, the refusals are described as having been initiated by the patients’ relatives and not the patients themselves. Nursing students and medical interns experience patient refusals e.g. patients state, "I do not want an Arab to touch me or take care of me. By contrast, Arab senior physicians or managers rarely encounter refusals.    **Consequences** - Very angry, sense of humiliation, haunting experiences. Insulted, hurt and perceived negative effect on ability to continue work. |
| 27 | Rhead et al., 2020^22^ | United Kingdom (UK) | Examine the impact of harassment and discrimination on NHS staff working in London trusts, utilising data from the 2019 TIDES cross-sectional survey. | 931 Medical/dental, allied health professionals/ psychological therapists, nurses, healthcare assistants and other professions (service managers, discharge coordinators, patient outcomes manager and medical secretaries;  **White Other (n=107; 11.5%), Black (n=179; 19.2%; 21.6%), Asian (n=166; 17.8%; 20.5%), Mixed (n=57; 6.1%; 3.5%), Migrant status**: **Migrant (n=328; 35.2%; 37.2%)** | Quantitative; Cross-sectional survey design | **Experiences** - Women, Black ethnic minority staff, migrants, nurses and healthcare assistants were most at risk of discrimination and/or harassment. 21% of the participants experienced discrimination and 44% experienced BHA from colleagues. HCPs from Black ethnic groups were more likely to experience and witness both discrimination and BHA compared with the White British group. Asian HCPs were also proportionately more likely to experience discrimination compared with White British respondents. Migrants were proportionately more likely to witness, and experience discrimination and experience harassment compared with non-migrants. Ethnicity and migrant status also factor into these findings, for example, healthcare assistants had the greatest proportion of migrants (56%) and non-white ethnic groups (62%) compared with any other HCP role. Nurses who reported discrimination mostly belonged to the Black ethnic group (38%).    **Consequences** - Experiencing BHA and witnessing discrimination were associated with moderate or severe somatic symptoms and lower job satisfaction was associated with experienced discrimination. Experiencing discrimination doubled the odds of having taken a minimum of 2 weeks sickness absence in the past year compared to those that had not. Experiencing BHA or discrimination was associated with probable anxiety or depression and somatic symptoms. Experiencing BHA was associated with moderate to severe somatic symptoms. Witnessing discrimination was associated with moderate to severe somatic symptoms but not probable anxiety or depression. Other - Nurses, healthcare assistants and other professions were more likely to experience discrimination than medical staff. |
| 28 | Ruvalcaba et al., 2018^29^ | United States (US) | To examine and describe whether differences and relationships existed between ESL and non-ESL nursing students perceptions of staff nurse incivility during their clinical rotations | 975 Nursing students; not specified (n=185, 19% **English as a second language**) | Quantitative; Cross-sectional survey design | **Experiences** - The majority of nursing students reported that they encountered uncivil behaviours either occasionally or rarely. Non-ESL nursing students perceived exclusionary behaviours and total incivility behaviours by staff nurses more frequently than did ESL nursing students. However, the effect sizes of those differences were minimal and did not provide enough evidence that ESL and non-ESL nursing students perceived staff nurses’ incivility differently. Female nursing students scored higher in incivility than male nursing students. ESL nursing students scored higher in vertical collectivism scale than did non-ESL students, indicating it is possible that ESL students’ values help them manage encounters with incivility in the clinical setting.    **Consequences** - Not stated |
| 29 | Srinivasa et al., 2020^26^ | United States (US) | The objective of this study was to give voice to the experience of minority nurses who self-identified as being bullied at work in acute care settings by understanding their experience. | 18 Nurses; **African American (n=7, 39%) Asian/Asian American (n=3, 17%) Hispanic/Hispanic American (n=5, 28%) Native American (n=1, 6%) Other (English as a Second Language) (n=2, 11%)** | Qualitative; Semi-structured interviews and survey data | **Experiences** - Participants frequently spoke of race or ethnic-based cliques and being ignored. False accusations and a lack of support for staff reporting bullying, gaslighting from managers that listen to certain groups with power over others. False accusations from supervisors and discussed privately by colleagues behind the nurses back. Deliberately calling staff members the wrong name, jokes about accents, race related jokes and making fun out of staff. Preceptor was mean and nasty.    **Consequences** - Disheartened and discouraged, 15 out of 18 participant reported anxiety and stress, from nervousness to anxiety and stress about getting things right and being perfect. Psychological responses include dry mouth, heart racing. Feeling fed up, dehumanised and demoralised. Sad, frustrated and other negative emotions, and crying heavily. Employee silence and intention to leave the job. |
| 30 | Steadman et al., 2009^23^ | United Kingdom (UK) | To determine the prevalence of bullying and experience of bullying behaviours among postgraduate hospital dentists. | 136 Postgraduate Hospital Dentists (different grades); 24% (32) were from **other ethnic groups.** | Quantitative; Cross sectional survey design | **Experiences** - No statistically significant difference in experience of bullying behaviours by gender or ethnic group though non-white participants were significantly more likely than white participants to have experienced four of the bullying behaviours e.g. inappropriate jokes, violence to property, unreasonable refusal of applications for leave, training or promotion, racial or sexual discrimination. Three of these behaviours fell into the category that posed a threat to the respondent’s personal standing.    **Consequences** - Not stated. |
| 31 | Thrasher et al., 2016^24^ | United States (US) | To examine the independent effect of workplace discrimination on alcohol abuse by including other measures of psychosocial stress and explicitly hypothesizing an interaction effect between minority status and reports of workplace discrimination. | 664 Administrator and professional (n=74, 11%), nursing (n=246, 37%) other clinical (n=93, 14%) (e.g., mental health, nursing assistants, rehabilitation), clerical (n=57, 9%), technical (n=139, 21%) and support (n=55, 8%) positions; **Asian/Pacific Islander 105 (16%) African American 185 (28%) Latino 66 (10%) Other/Mixed 48 (7%)** | Quantitative; Cross-sectional survey design | **Experiences**: Workplace discrimination was associated with race or ethnicity (with the Black and White ethnic groups being most likely to report these experiences).    **Consequences** - Not stated. |
| 32 | Ulusoy & Schablon 2021^27^ | Germany | The aim of the study was to investigate whether care workers with Turkish migration background in in-patient geriatric care are exposed to discrimination from residents. | 24 Care workers: (Certified (healthcare) nurse in senior position (n=4, 16.7%) Certified (healthcare) nurse, not in senior position (n=3, 12.5%) Geriatric nurse/healthcare nurse (n=6, 25.0%) Nurse without formal qualification (n=3, 12.5%) Trainee (n=2, 8.3%) Other (catering service, ward service/kitchen assistant, gap year volunteers, etc.) (n=6, 25.0%); **Turkish heritage** | Qualitative; Semi-structured interviews | **Experiences** - Personal and witnessed experience of discrimination. Respondents reported events where the residents refused to receive care from them both verbally (“I don’t want you” and non-verbally (signalling an unwillingness to be touched). Rejections expressed in non-verbal form through body language e.g. being ignored or refusing to be touched by the nurse. Language and communication barriers. Verbal attacks by patients that insinuated incompetence and making accusatory remarks.    **Consequences** - Highly stressful, difficulties providing nursing care. |

Definitions: NCT – Nursing Care Technician; PCT- Patient Care Technician; SAs- Support Assistant; RNs- Registered Nurses; ProQOL - professional quality of life; ESL – English as a Second Language; BHA – Bullying Harassment and Abuse; WPV – Workplace Violence; BAME – Black, Asian, Minority Ethnic; HCP – Healthcare Professional

**References (in text citation order)**

1. Alshehry, A.S., Alquwez, N., Almazan, J., Namis, I.M., Moreno-Lacalle, R.C., and Cruz, J.P*.* (2019) 'Workplace incivility and its influence on professional quality of life among nurses from multicultural background: A cross‐sectional study,' *Journal of Clinical Nursing* [Preprint]. <https://doi.org/10.1111/jocn.14840>.
2. Bae, S.H., Dang, D.D., Karlowicz, K.A., and Kim. M. (2020) 'Triggers contributing to health care clinicians’ disruptive behaviors,' *Journal of Patient Safety*, 16(3), pp. e148–e155. <https://doi.org/10.1097/pts.0000000000000288>.
3. Bagilhole, B. and Stephens, M. (1999) 'Management responses to equal opportunities for ethnic minority women within an NHS hospital trust,' *Journal of Social Policy* [Preprint]. <https://doi.org/10.1017/s0047279499005541>.
4. Brooks, M.E. (2016) 'Voices of perseverance: A phenomenological exploration of the life histories of female African-American registered nurses,' *Thesis; Eastern Michigan University* [Preprint]. <https://commons.emich.edu/cgi/viewcontent.cgi?article=2176&context=theses>.
5. Brunton, M. and Cook, C. (2018) 'Dis/Integrating cultural difference in practice and communication: A qualitative study of host and migrant Registered Nurse perspectives from New Zealand,' *International Journal of Nursing Studies*, 83, pp. 18–24. <https://doi.org/10.1016/j.ijnurstu.2018.04.005>.
6. Cohen, B. (2013) *Torah true: The lived experience of orthodox Jewish registered nurses*. PhD Thesis. The City University of New York.
7. Cottingham, M.D., Johnson, A.H. and Erickson, R.J. (2018) '“I can never be too comfortable”: race, gender, and emotion at the hospital bedside,' *Qualitative Health Research*, 28(1), pp. 145–158. <https://doi.org/10.1177/1049732317737980>.
8. Darr, A. (2017) 'Palestinian Arabs and Jews at work: Workplace encounters in a War-Torn country and the grassroots strategy of ‘Split Ascription,’' *Work, Employment & Society*, 32(5), pp. 831–849. <https://doi.org/10.1177/0950017017711141>.
9. Deery, S., Walsh, J. and Guest, D. (2011) 'Workplace aggression: the effects of harassment on job burnout and turnover intentions,' *Work, Employment & Society*, 25(4), pp. 742–759. <https://doi.org/10.1177/0950017011419707>.
10. Dreachslin, J.L., Hunt, P.L. and Sprainer, E. (2000) 'Workforce diversity: implications for the effectiveness of health care delivery teams,' *Social Science & Medicine*, 50(10), pp. 1403–1414. <https://doi.org/10.1016/s0277-9536(99)00396-2>.
11. Floyd, C. (2020) 'Empowering nurses of minority in the face of incivility and bullying: Through the lens of phenomenology,' [*www.academia.edu*](http://www.academia.edu) [Preprint]. <https://www.academia.edu/82764880/Empowering_Nurses_of_Minority_in_the_Face_of_Incivility_and_Bullying_Through_the_Lens_of_Phenomenology>.
12. Jia, H., Fang, H., Chen, R., Jiao, M., Wei, L., Zhang, G., Li, Y., Wang, Y., Wang, Y., Jiang, K., Li, J., Jia, X., Ismeal, O.Y., Mao, J and Wu, Q. (2020) 'Workplace violence against healthcare professionals in a multiethnic area: a cross-sectional study in southwest China,' *BMJ Open*, 10(9), p. e037464. <https://doi.org/10.1136/bmjopen-2020-037464>.
13. Johnson, J., Cameron, L., Mitchinson, L., Parmar, M., Opio-Te, G., Louch, G., and Grange, A. (2019) 'An investigation into the relationships between bullying, discrimination, burnout and patient safety in nurses and midwives: is burnout a mediator?,' *Journal of Research in Nursing*, 24(8), pp. 604–619. <https://doi.org/10.1177/1744987119880329>.
14. Keshet, Y. and Popper-Giveon, A. (2016) 'Work experiences of ethnic minority nurses: a qualitative study,' *Israel Journal of Health Policy Research*, 5(1). <https://doi.org/10.1186/s13584-016-0076-5>.
15. Keshet, Y. and Popper-Giveon, A. (2017) 'Race-based experiences of ethnic minority health professionals: Arab physicians and nurses in Israeli public healthcare organizations,' *Ethnicity & Health*, 23(4), pp. 442–459. <https://doi.org/10.1080/13557858.2017.1280131>.
16. Keshet, Y., Raviv, B., Popper-Giveon, A., Strizhevski, A., and Abu-Kella, A. (2017) 'Tensions and Coping Strategies in Ethnically Mixed Teams: Findings from a Study in Two Emergency Departments,' *Journal of Immigrant and Minority Health*, 20(4), pp. 951–962. <https://doi.org/10.1007/s10903-017-0603-7>.
17. Larsen, J. (2007) 'Embodiment of discrimination and overseas nurses’ career progression,' *Journal of Clinical Nursing*, 16(12), pp. 2187–2195. <https://doi.org/10.1111/j.1365-2702.2007.02017.x>.
18. Mitchell, S.C. (2020) *The Lived Experiences of African American Nurses Transitioning to the Workforce*. PhD Thesis. Walden University.
19. Moceri, J.T. (2012) 'Bias in the nursing workplace: implications for Latino(a) nurses.,' *PubMed*, 19(3), pp. 94–101. <https://pubmed.ncbi.nlm.nih.gov/23155895>.
20. Popper-Giveon, A. and Keshet, Y. (2020) 'Workforce diversity in the context of violent conflict: Public hospitals in Israel,' *Human Service Organizations, Management, Leadership & Governance*, 45(1), pp. 66–78. <https://doi.org/10.1080/23303131.2020.1855282>.
21. Popper-Giveon, A. and Keshet, Y. (2018) 'The secret Drama at the Patient’s Bedside—Refusal of treatment because of the practitioner’s ethnic identity: the medical Staff ’s point of view,' *Qualitative Health Research*, 28(5), pp. 711–720. <https://doi.org/10.1177/1049732318755676>.
22. Rhead, R., Chui, Z., Bakolis, I., Gazard, B., Harwood, H., MacCrimmon, S., Woodman, C., and Hatch, S. (2020) 'Impact of workplace discrimination and harassment among National Health Service staff working in London trusts: results from the TIDES study,' *British Journal of Psychiatry Open*, 7(1). <https://doi.org/10.1192/bjo.2020.137>.
23. Steadman, L., Quine, L., Jack, K., Felix, D.H., and Waumsley, J. (2009) 'Experience of workplace bullying behaviours in postgraduate hospital dentists: questionnaire survey,' *British Dental Journal*, 207(8), pp. 379–380. <https://doi.org/10.1038/sj.bdj.2009.901>.
24. Thrasher, A.D., Wells, A.M., Spencer, M.S., Cofie, L., and Yen, I.H. (2016) 'Workplace discrimination is associated with alcohol abuse among ethnically diverse hospital staff,' *AAOHN Journal*, 64(5), pp. 202–209. <https://doi.org/10.1177/2165079916628878>.
25. Moceri, J.T. (2013) 'Hispanic nurses’ experiences of bias in the workplace,' *Journal of Transcultural Nursing*, 25(1), pp. 15–22. <https://doi.org/10.1177/1043659613504109>.
26. Srinivasa, E. (2020) *Experiences of Self-Reported Bullying in Minority Nurses within Acute Care Hospital Workplace Settings*. <https://scholarworks.umb.edu/doctoral_dissertations/634/>.
27. Ulusoy, N. and Schablon, A. (2020) 'Discrimination in In-Patient Geriatric Care: A Qualitative Study on the Experiences of Employees with a Turkish Migration Background,' *International Journal of Environmental Research and Public Health*, 17(7), p. 2205. <https://doi.org/10.3390/ijerph17072205>.
28. Iheduru-Anderson, K., Agomoh, C.J. and Inungu, J. (2021) 'African born black nurses’ perception of their U.S. work environment: Race matters,' *Nursing Outlook*, 69(3), pp. 409–424. <https://doi.org/10.1016/j.outlook.2020.11.009>.
29. Ruvalcaba, J.G., Welch, S. and Carlisle, J. (2018) 'ESL Versus Non-ESL Nursing Students’ Perceptions of Incivility in the Clinical Setting,' *Journal of Nursing Education*, 57(12), pp. 720–726. <https://doi.org/10.3928/01484834-20181119-04>.
30. Onyeibe, C.J. (2021) *Experience of native ethnic African minority nurses working in United States public hospitals - ProQuest*. PhD Thesis. Capella University. <https://www.proquest.com/openview/607c06730c05d99743ffce78ed72d3b1/1?pq-origsite=gscholar&cbl=18750&diss=y>.
31. Pierce, L. (2018) *Exploring The Experiences Of African American Nurses: An Emancipatory Inquiry*. PhD thesis. University of North Dakota.
32. Boateng, G.O. and Adams, T.L. (2016) '“Drop dead … I need your job”: An exploratory study of intra-professional conflict amongst nurses in two Ontario cities,' *Social Science & Medicine*, 155, pp. 35–42. <https://doi.org/10.1016/j.socscimed.2016.02.045>.
